# Clinical Potential of Novel Microbial Therapeutic LP51 Based on Xerosis-Microbiome Index

**DOI:** 10.3390/cells13232029

**Published:** 2024-12-09

**Authors:** Sukyung Kim, Md Abdur Rahim, Hanieh Tajdozian, Indrajeet Barman, Hyun-A Park, Youjin Yoon, Sujin Jo, Soyeon Lee, Md Sarower Hossen Shuvo, Sung Hae Bae, Hyunji Lee, Sehee Ju, Chae-eun Park, Ho-Kyoung Kim, Jeung Hi Han, Ji-Woong Kim, Sung geon Yoon, Jae Hong Kim, Yang Gyu Choi, Saebim Lee, Hoonhee Seo, Ho-Yeon Song

**Affiliations:** 1 Human Microbiome Medical Center (HM·MRC), Soonchunhyang University, 22, Soonchunhyang-ro, Sinchang-myeon, Asan-si 31538, Chungnam, Republic of Korea; microsk@sch.ac.kr (S.K.); abdurrahimju103@gmail.com (M.A.R.); hanieh.mahsa75@gmail.com (H.T.); tapanindrajeet@gmail.com (I.B.); ttll2915@naver.com (H.-A.P.); dbsdbwls966@gmail.com (Y.Y.); sujiny0206@gmail.com (S.J.); sarowerhossen9@gmail.com (M.S.H.S.); sunghae@sch.ac.kr (S.H.B.); hyunji@sch.ac.kr (H.L.); joosehui@sch.ac.kr (S.J.); cheun95@naver.com (C.-e.P.); ghrud3759@hanmail.net (H.-K.K.); saevim@hanmail.net (S.L.); 2 Department of Microbiology and Immunology, School of Medicine, Soonchunhyang University, 31, Suncheonhyang 6-gil, Dongnam-gu, Cheonan-si 31151, Chungnam, Republic of Korea; isoyeon517@gmail.com; 3 Materials Science Research Institute, LABIO, Inc., 184 Gasan Digital 2-ro, Geumcheon-gu, Seoul 08501, Republic of Korea; msri05@labio.kr (J.H.H.); msri12@labio.kr (J.-W.K.); msri16@labio.kr (S.g.Y.); msri06@labio.kr (J.H.K.); msri22@labio.kr (Y.G.C.)

**Keywords:** microbiome therapeutic LP51, vaginal microbiota, xerosis, xerosis-microbiome index, *Firmicutes*/*Actinobacteria* ratio

## Abstract

Xerosis, characterized by dry, rough skin, causes discomfort and aesthetic concerns, necessitating effective treatment. Traditional treatments often show limited efficacy, prompting the need for innovative therapies. This study highlights the efficacy of microbiome therapeutic LP51, derived from a healthy vaginal microbiome, in improving xerosis. A double-blind clinical trial involving 43 subjects with dry inner arm skin compared the effects of a 2.9% LP51 extract formulation to a placebo over 4 weeks. The LP51 group exhibited a significant increase in stratum corneum hydration (10.0 A.U.) compared to the placebo group (4.8 A.U.) and a 21.4% decrease in transepidermal water loss (TEWL), whereas the placebo group showed no significant change. LP51 also demonstrated benefits in enhancing skin hydration, improving the skin barrier, and exhibited anti-atopic, anti-inflammatory, and antioxidant properties. Safety was confirmed through in vitro cytotoxicity tests. These effects are attributed to the microbiome-safe component in LP51 and its role in improving xerosis, reflected by an increase in the xerosis-microbiome index, defined by the Firmicutes/Actinobacteria ratio. These findings position microbiome therapeutic LP51 as a promising novel treatment for xerosis.

## 1. Introduction

The skin is the largest organ of the human body, functioning as a defense barrier between the body and the environment. It regulates the movement of water and electrolytes, reduces penetration by harmful chemicals, prevents the entry of external antigens and microbial pathogens, and protects skin cells from ultraviolet radiation [1,2]. Additionally, the skin plays a critical role in maintaining body homeostasis, particularly in terms of composition, heat regulation, blood pressure control, and excretory functions [1]. Therefore, maintaining healthy skin is essential for daily activities, while dysfunction can lead to various skin disorders and negatively affect quality of life.

The stratum corneum, a water-retaining layer of the epidermis, serves as a highly efficient barrier against water loss under most conditions [3]. However, this barrier is constantly exposed to desiccation stress due to its proximity to the environment and is frequently damaged by external factors, leading to its breakdown and resulting in dry skin or xerosis [3]. Clinicians describe dry skin as rough, scaling, and potentially cracked, often linked to conditions such as chapping, eczema, seborrheic dermatitis, psoriasis, pruritus, and atopic dermatitis, all of which share symptoms such as scaling, flaking, and roughness [4]. Conventional therapies for these conditions are primarily chemical based and may often have limited efficacy, with occasional potential side effects [5]. Given these concerns, clinicians and scientists are developing natural-based skin treatments that can improve skin conditions with minimal or no side effects [3]. In this context, microbiome-based therapeutics present a promising option, as their beneficial effects on skin health are well-documented [6,7].

Maintaining a balanced skin microbiota is crucial for healthy skin and is also linked to beauty [8,9]. Disruption of this balance, such as through dry skin, is associated with various skin disorders [4]. Studies have shown that microbiome-based therapeutics, such as the application of specific probiotic strains and their byproducts in formulations, have therapeutic and cosmetic effects, improving various skin conditions and enhancing overall skin health [10]. Recent clinical trials with strains such as *Lactobacillus plantarum*, *L. casei*, *L. rhamnosus*, and *Pediococcus acidilactici* have demonstrated their potential to improve skin conditions and maintain a balanced skin microbiota [7,11,12].

Building on these findings, we conducted a 4-week double-blind clinical trial involving 43 participants suffering from xerosis to evaluate the therapeutic potential of Microbiome therapeutic LP51. Prior to the clinical trial, we confirmed the efficacy of this strain through in vitro tests. The clinical trial demonstrated significant improvements in skin conditions, along with enhanced skin microbiota. This study aimed to develop a microbiome-based therapeutic using Microbiome therapeutic LP51 for managing and treating dermatological disorders.

## 2. Materials and Methods

### 2.1. Culturomics-Driven Isolation of Candidate Strains from Healthy Vaginal Fluid

*Lactobacillus* strains were isolated from healthy vaginal microbiota using a culturomics approach combined with 16S rRNA sequencing technology. This study specifically focused on *Lactobacillus* strains, as previous NGS metagenomics analyses identified them as key biomarkers in healthy vaginal samples [13,14]. The procedure for collecting specimens from healthy Korean women was detailed in our previous research [13,14]. In brief, preserved vaginal swab samples were freeze-thawed, diluted, and spread onto agar media, including modified MRS agar containing 0.12 g/L of bromocresol purple (Sigma Aldrich, Steinheim, Germany) and 0.1% L-cystine HCl (Sigma Aldrich, Germany), as well as Rogosa SL agar. Cultures were incubated at 37 °C for 0–48 h under varying conditions—namely aerobic, anaerobic, and microaerophilic chambers (Baker Ruskin, Rotherham, UK). After incubation, plates were inspected, and colonies were selected based on size, shape, color, and morphology. Selected colonies were sub-cultured to obtain pure isolates. Once pure colonies of interest were obtained, they were cultured in the appropriate broth medium and sent to a commercial sequencing company for identification. Concurrently, the isolates were preserved at −80 °C in a 30% glycerol solution for future use.

### 2.2. 16S rRNA Gene-Sequencing-Based Identification of Isolated Probiotics

The probiotic strain was identified through 16S rRNA gene-sequencing technology. After DNA extraction, amplification was performed via PCR using the primer pair 27F (5-AGA GTT TGA TCC TGG CTC AG-3) and 1492R (5-GGT TAC CTT GTT ACG ACT T-3). An ABI PRISM 3730XL DNA analyzer (Applied Biosystems, Waltham, MA, USA) was then used for purification and sequencing of the amplified PCR product. The resulting sequencing data were compared with entries in the National Center for Biotechnology Information (NCBI) GenBank database using BLAST (basic local alignment search tool) to identify the strain.

### 2.3. Whole-Genome Sequencing of Probiotic Strain

Whole-genome sequencing (WGS) was performed to identify the candidate probiotic strain at the strain level. Genomic DNA was extracted using a QIAamp DNA Extraction Kit (Qiagen, Germany) according to the manufacturer’s instructions and sent to a commercial WGS service company (Chunlab Inc., Republic of Korea). SMRTLink (13.1.0) was used to compile the PacBio sequencing data using the HGAP2 protocol. The resulting contigs were circularized using Circlator 1.4.0 (Sanger Institute Hinxton, UK). Prodigal 2.6.2 was used to predict protein-coding sequences (CDSs), which were classified according to orthologous groups (EggNOG 4.5; http://eggnogdb.embl.de, accepted 22 November 2024). tRNAs were identified using tRNAscan-SE 1.3.1, and rRNAs and other noncoding RNAs were identified through covariance model searches in the Rfam 12.0 database. The OrthoANIu-algorithm-based Average Nucleotide Identity (ANI) calculator was used to compare the prokaryotic genome sequences.

### 2.4. Preparation of Master Cell Bank (MCB) and Working Cell Bank (WCB)

The Master Cell Bank (MCB) for the probiotic strain was prepared according to the ICH Q5D-compliant GMP manufacturing guidelines, recognizing that the quality of a biological product depends on the quality of the cells used in its production [15]. After bacterial cell identification, the MCB preparation was initiated. Prior to beginning the process, the biosafety cabinet was thoroughly cleaned and decontaminated according to standard protocols. The bacterial strains were aseptically cultured under previously mentioned conditions and then transferred into cryovials containing 60% autoclaved glycerol. The cryovials were shaken for 5 min to ensure thorough mixing of the bacterial culture with the glycerol. Working Cell Banks (WCBs) were subsequently prepared from the MCB under defined culture conditions. Quality control tests were performed to confirm the genetic identity of the MCB and WCB and to ensure the WCB was free from contaminants. Both the MCB and WCB were stored at −80 °C for future use.

### 2.5. Cultivation of LP51 Using a Lab-Scale Fermenter with Food-Grade Media (FGM)

*Lactobacillus rhamnosus* LABIO-PMC-51 (Microbiome therapeutic LP51) was cultivated in two different types of food-grade media (FGM) for optimization, with the aim to develop the strain as a potential therapeutic agent. Both FGMs were based on MRS medium but contained only non-animal components. The initial pH of the FGM was adjusted from 7.5 to 6.3 using 6 N HCl. Before being scaled up, the probiotic strain was cultured in 30 mL of FGM and incubated at 37 °C for 24 h. Once bacterial growth was confirmed, the strain was transferred to a lab-scale fermenter (Fermentec, FMT-ST-S07, Cheongju, Republic of Korea) containing 4 L of FGM. The fermenter was autoclaved at 121 °C for 15 min. After reaching the optimal temperature, 1.0% of the probiotic culture was aseptically added and fermented at 37 °C for 43 h using a fermenter impeller. Bacterial growth and pH were monitored at 0, 4, 20, 24, 28, and 43 h using a spectrophotometer (HACH, DR 1900, Loveland, CO, USA) and a pH meter (Mettler-Toledo, Greifensee, Switzerland). The strain showing the best growth in the medium with lower pH was selected for further processing. The culture was centrifuged at 4000 rpm for 30 min, and the supernatant was filtered using a 0.22 μm membrane filter (Millipore, Waltham, MA, USA). The resulting culture filtrate was used for subsequent in vitro experiments. The supernatant under these conditions was taken as 100%, and this supernatant was diluted with medium and applied to in vitro experiments.

### 2.6. Confirmation of HAS3 (Hyaluronan Synthase 3), FLG (Filaggrin), IVL (Involucrin), and LOR (Loricrin) Expression

The effect of LP51 culture filtrate on skin barrier function and moisturizing properties was evaluated using human keratinocyte cells (HaCaT). HaCaT cells were cultured in DMEM (Dulbecco’s Modified Eagle’s Medium) (Welgene, Gyeongsan, Republic of Korea) supplemented with 10% FBS (fetal bovine serum) (Welgene, Republic of Korea) and 1% penicillin/streptomycin (Gibco, Waltham, MA, USA) and maintained in a humidified atmosphere with 5% CO_2_ at 37 °C. Cells were then seeded at 5 × 10^5^ cells/mL in a 6-well plate. After overnight incubation, the medium was replaced with serum-free medium, and the cells were incubated for an additional 24 h. Next, cells were treated with 0.75%, 1.5%, and 3% dilutions of LP51 culture filtrate for 6 h and washed with DPBS (Dulbecco’s phosphate-buffered saline) (Welgene, Republic of Korea), and RNA was extracted using the TaKaRa Mini BEST Universal RNA Extraction Kit (Takara, Kusatsu, Japan). At this time, 1 µg/mL of Human Epidermal Growth Factor (hEGF, JForCell, JFC-GF008, Daejeon, Republic of Korea) was used as the positive control. After quantification of the RNA concentration, cDNA was synthesized, and real-time PCR was performed using the TaqMan master mix on a StepOnePlus Real-Time PCR System (Applied Biosystems, Forster city, CA, USA). The expression of HAS3 and FLG was then analyzed. TaqMan primers for HAS3 (Hs00193436_m1), FLG (Hs01566408_m1), and GAPDH (Hs02758991_g1) were obtained from Life Technologies, Waltham, MA, USA. Additionally, the expression of IVL and LOR [16] was explored using a SYBR green reagent (BioRad, Hercules, CA, USA).

### 2.7. Determining Thymus and Activation-Regulated Chemokine (TARC) Production

The anti-atopic effect of the LP51 culture filtrate was evaluated using HaCaT cells. The cells were cultured in a 24-well plate until a confluent monolayer was formed and then treated with a combination of tumor necrosis factor-α (TNF-α, R&D Systems, 210-TA-005/CF, Minneapolis, MN, USA) at 10 ng/µL, interferon-γ (IFN-γ, 285-IF-100/CF, R&D Systems, USA) at 20 µg/mL, and dexamethasone (D4902, Sigma-Aldrich, St. Louis, MO, USA) at 10 µg/mL, and 0.25%, 0.5%, 1% of LP51 culture filtrate, followed by an additional 24-h incubation. After incubation, the culture media were collected and centrifuged, and the supernatant was harvested. The supernatant was used to measure the production of TARC using an ELISA Kit (R&D Systems, USA).

### 2.8. Assessing Nitric Oxide (NO) Generation

The anti-inflammatory effect of *LP51* culture filtrate was assessed using RAW 264.7 macrophage cells. Cells were seeded at 2.5 × 10^5^ cells/well in a 96-well plate and incubated overnight in a humidified atmosphere with 5% CO_2_ at 37 °C. After overnight incubation, the medium was replaced with serum-free medium and incubated for an additional 24 h. Cells were then treated with 0.25%, 0.5%, and 1% of LP51 culture filtrate; 20 µM of quercetin (Q4951, Sigma, USA); and/or 1 µg/mL of lipopolysaccharides (LPS, L4391, Sigma, USA) for 24 h. Afterward, 100 µL of the culture medium from treated cells was transferred to a fresh 96-well plate, treated with Griess reagent (Sigma-Aldrich, St. Louis, MO, USA), and incubated for 20 min at room temperature. The plate was read at 540 nm using a multiplate reader (Perkin Elmer, Waltham, MA, USA) to quantify the synthesized NO.

### 2.9. Exploring the DPPH Scavenging Capacity

The antioxidant effect of LP51 culture filtrate was assessed using the 2,2-diphenyl-1-picrylhydrazyl (DPPH) assay (Sigma-Aldrich, USA) as previously described [17]. Briefly, 100 µL of 0.2 mM DPPH solution was mixed with 100 µL of diluted LP51 culture filtrate (at concentrations of 1.875%, 3.75%, 7.5%, 15%, and 30%) or 50 µg/mL of L-ascorbic acid (vitamin C) and incubated for 30 min in the dark at room temperature. For the control group, DPPH was combined with the same volume of ethanol without any test substance. After incubation, absorbance was measured at 520 nm using an ELISA reader (Thermo Labsystems, Basingstoke, UK), and the DPPH scavenging activity was calculated.

### 2.10. Evaluating the In Vitro UV Protection Ability

The UV protection ability of LP51 culture filtrate was evaluated using human keratinocyte HaCaT cells. Cells were seeded at 1 × 10^4^ cells/well in a 96-well black plate and incubated overnight. After incubation, the cells were washed with HBSS (Hank’s Balanced Salt Solution) and treated with various concentrations of LP51 culture filtrate for 24 h. The cells were then exposed to UVB irradiation at 20 mJ/cm^2^, followed by an additional 24-h incubation. Cell viability was measured using the EZ-Cytox cell viability assay kit (DoGenBio, Seoul, Republic of Korea).

### 2.11. Preparation of Test Formulation Using LP51 Culture Filtrate for Clinical Trial

The LP51 formulation was prepared using the LP51 culture filtrate, as described in previous sections, as the primary ingredient to investigate its efficacy on dry skin. The test formulation consisted of water (86.55%), glycerin (3%), carbomer (0.4%), xanthan gum (0.05%), caprylic/capric triglyceride (1%), cetyl alcohol (0.7%), glyceryl stearate (0.7%), PEG-75 stearate (0.3%), ceteth-20 (0.15%), steareth-20 (0.15%), 1,2-hexanediol (2.1%), propanediol (2%), and LP51 culture filtrate (2.9%). In the control group of the clinical trial, a placebo cream was prepared with the same ingredients except for the active ingredient, LP51 culture filtrate. The concentration of LP51 was selected to effectively demonstrate efficacy while preserving the viscosity and integrity of the formulation, with observations conducted across various concentrations to ensure optimal performance.

### 2.12. Stability Test of the Prepared Formulation

The formulated product was subjected to thermal stability tests at various temperatures, as described previously [18]. The product was stored at chilled temperature (4 °C), room temperature (23 ± 2 °C), elevated temperature (45 °C), and a cycling temperature of 4 °C → 25 °C → 45 °C, where the product was kept at each temperature for 8 h during the cycle. During storage, the phase, odor, and color of the product were observed at 0, 1, 2, 4, 9, and 11 weeks.

### 2.13. Selection Criteria for Test Subjects in the LP51 Clinical Trial for Dry Skin

A 4-week clinical trial was conducted to evaluate the impact of LP51 culture filtrate on dry skin, enrolling 46 Korean adults (both male and female) who met the inclusion criteria and did not fall under the exclusion criteria. The inclusion criteria required participants to be healthy men and women aged 19–70 years without acute or chronic diseases (excluding atopic dermatitis), individuals suffering from dry skin and itchiness in the test area (crook of the arm), those with objectively proven skin barrier damage demonstrated by a transepidermal water loss (TEWL) value of ≥12 g/h/m^2^ and a skin hydration value below 35 (Corneometer measurement), individuals who provided voluntary informed consent, and those who agreed to comply with study visits and observations. The exclusion criteria ruled out individuals with active skin diseases requiring treatment or those with an ESIF (erythema, scaling, induration, fissures) score > 6; individuals with a history of antibiotic, steroid, immunosuppressant, antihistamine, or retinoid use, or phototherapy within the previous 4 weeks; individuals who used skin-disease-related supplements or moisturizers within 2 weeks; those participating in other clinical trials within the previous 4 weeks; individuals with frequent exposure to UV light; pregnant or lactating women or those planning pregnancy during the trial period; and those with any other factors deemed unsuitable by the investigator. This study was conducted in adherence to the ethical principles outlined in the Declaration of Helsinki (1964).

### 2.14. Study Design for the Clinical Trial

The effect of Lactobarriome 5% cream containing LP51 culture filtrate on dry skin was evaluated in a 4-week double-blind clinical trial. Participants were screened and selected based on the inclusion and exclusion criteria. A random allocation chart (simple randomization method) was used to assign participants to the experimental group (test formulation) or control group (placebo). The volunteers applied the product twice daily (morning and evening) to a 3 cm area proximal and distal to the crook of the designated arm. Clinical parameters were evaluated at baseline, after 2 weeks, and after 4 weeks of use. Visual assessments, subjective pruritus evaluations, skin hydration measurements, and TEWL measurements were conducted at every visit by a skin professional. Additionally, magnified photographs were taken using a Folliscope, and skin samples were collected to assess microbial changes after treatment. Prior to testing, the designated arm was washed with water and allowed to relax for 30 min under constant conditions (22 ± 2 °C, 50 ± 10% RH).

### 2.15. Evaluation of Skin Hydration, Transepidermal Water Loss, Subjective Pruritus Severity, and Visual Appearance

Skin hydration was measured using a Corneometer (Courage and Khazaka, Köln, Germany) at baseline and after 2 and 4 weeks. The device was lightly pressed onto the test area, and hydration was measured 3 times with a 5 s interval between each measurement, assessing a depth of 10–20 μm below the stratum corneum. TEWL was measured to evaluate the effect of LP51 on the keratin layer’s permeability. A Tewameter (Courage + Khazaka electronic GmbH, Köln, Germany) was used, with the probe being pressed against the test area with constant pressure for 30 s. The measurement was repeated 3 times, and the average of the final three values was recorded. Subjective pruritus was assessed using a 10 cm visual analogue scale (VAS), where subjects rated their itchiness. Scores ranged from 0 (no pruritus) to 10 (very severe pruritus). Visual assessments of erythema, scaling, induration, and fissuring (ESIF) were conducted at each visit by a dermatology specialist, with a total score of 0–12. Photographs of the tested sites were also taken using a digital camera (Canon, Tokyo, Japan) and Folliscope (LeadM, Seoul, Republic of Korea).

### 2.16. Skin Metagenomics Analysis

To evaluate the impact of the test formulation on microbial composition changes in the treated skin, 16S-rRNA-based amplicon sequencing was performed. Briefly, after treatment, skin samples were collected using an NBgene-SKIN kit (Noble Bio, Hwaseong, Republic of Korea) via a gentle rubbing of the area within a 3 cm proximal range (3 × 3 cm^2^ test site) to the crook of the arm. The samples were transported to the Human Microbiome Medical Research Center (HM-MRC) and stored at −80 °C until further analysis. Skin DNA was extracted from the collected samples through mechanical lysis with bead beating. After sufficient centrifugation, the supernatant containing no visible particles was collected, and the quality of the extracted DNA was assessed using agarose gel electrophoresis. The DNA was also quantified using the Qubit dsDNA HS Assay Kit (Invitrogen, Waltham, MA, USA).

The V4 region of the 16S rRNA gene was then amplified using the Illumina 16S bacterial rRNA amplicon primer set (16S-F: TCGTCGGCAGCGTCAGATGTGTATAAGAGACAGCCTACGGGNGGCWGCAG, 16S-R: GTCTCGTGGGCTCGGAGATGTGTATAAGAGACAGGACTACHVGGGTATCTAATCC) (5 μM each), 10 ng of template DNA, and KAPA HiFi HotStart ReadyMix (Kapa Biosystems, Wilmington, MA, USA) according to a previously described protocol [13]. PCR was performed on all samples, including a negative control (no template DNA) and a positive control (5 ng of skin DNA) using a Veriti 96-well Thermal Cycler (Applied Biosystems, USA). The PCR conditions consisted of an initial denaturation at 95 °C for 3 min, followed by 25 cycles of denaturation at 95 °C for 30 s, annealing at 55 °C for 30 s, and extension at 72 °C for 30 s, with a final extension at 72 °C for 5 min. PCR products were purified using AMPure beads (Beckman Coulter, South Kraemer Boulevard Brea, CA, USA) according to the manufacturer’s instructions. Five microliters of the purified amplicon PCR product from each sample was used for indexing. A Nextera XT DNA Library Prep Kit (Illumina, San Diego, CA, USA) was employed for index PCR, followed by another clean-up using AMPure beads. The final DNA concentration of each sample was adjusted to 1 nM with H_2_O. Then, 5 μL of each sample was pooled together. The pooled library (50 pMol) was sequenced with 30% PhiX spiking using the iSeq100 platform (Illumina, USA).

The Quantitative Insights Into Microbial Ecology (QIIME) software (version 1.9.1) was used for sequence analysis [19]. Sequences were assigned to operational taxonomic units (OTUs) with a 97% similarity threshold. Representative sequences for each OTU were selected, and taxonomic data were assigned using the RDP classifier [20]. These representative sequences were then mapped to the taxonomy hierarchy, from phylum to family, based on the Human Microbiome Database. A Bayesian approach with a 97% confidence threshold was employed for taxonomic assignment. To assess the richness and diversity of bacterial populations within the samples, α-diversity indices, including observed OTUs, Chao1, ACE, Simpson, Shannon, and Fisher indices, were calculated [20,21,22,23]. The variation in bacterial communities between samples (β-diversity) was analyzed using indices such as Bray–Curtis, Jansen–Shannon, and Jaccard [24,25,26]. Permutational multivariate analysis of variance (PERMANOVA) was applied to determine the significance of beta diversity between the groups.

### 2.17. Determining the MIC and MBC of LP51 Against Skin Pathogens

To evaluate the antibacterial effectiveness of the LP51 culture filtrate, a minimal inhibitory concentration (MIC) and minimal bactericidal concentration (MBC) assay was conducted. The MIC and MBC were determined against skin pathogens, including *Staphylococcus aureus* KCTC 3881 and *Propionibacterium acnes* KCTC 3314, according to the guidelines of the Clinical and Laboratory Standards Institute (CLSI). Initially, *S. aureus* and *P. acnes* were cultivated at 37 °C for 24 h using tryptic soy broth and brain heart infusion broth, respectively. A 100 µL inoculum suspension containing the test substance was added to each well of a 96-well plate and incubated for 24 h. The MIC values were determined as the lowest filtrate concentrations that completely inhibited visible bacterial growth. After the MIC of the LP51 culture filtrate was determined, 20 µL aliquots from wells with no visible bacterial growth were spread onto the respective agar medium and incubated at 37 °C for 24 h. The MBC was defined as the lowest filtrate concentration showing no bacterial growth after incubation.

### 2.18. Cytotoxicity Test of LP51

The cytotoxicity of LP51 was evaluated on RAW 264.7 and HaCaT cells using an EZ-Cytox cell viability assay kit (DoGenBio, Republic of Korea) as previously described [27]. Briefly, the cells were grown in DMEM containing 10% FBS and 1% penicillin/streptomycin at 37 °C in a 5% CO_2_ incubator. RAW 264.7 and HaCaT cells (2.5 × 10^5^ cells/well) were seeded into a 96-well plate (SPL Life Sciences, Pocheon, Republic of Korea) and incubated overnight. Once the cells reached approximately 75–85% confluency, they were treated with 0.05, 0.5, and 1% of LP51 culture filtrate/media to the RAW264.7 cell and 1.5 and 3% of LP51 culture filtrate/media to the HaCaT cell for 24 h. Following incubation, 20 µL of WST solution was added to each well and incubated for an additional 2 h. Cytotoxicity was assessed by measuring the absorbance at 570 nm using a multiplate reader (Perkin Elmer, USA). The cytotoxicity of LP51 was also evaluated on HT29 cells using a lactate dehydrogenase (LDH) release assay according to a previously established protocol [28]. HT29 cells were cultured under the same conditions and seeded (1 × 10⁴ cells/well) into a 96-well plate. After overnight incubation, the cells were washed and treated with 1 × 10^7^, 1 × 10^8^, and 1 × 10^9^ CFU (colony forming unit)/well of LP51 for 24 h. The release of LDH into the medium was measured using a cytotoxicity detection kit (Roche Diagnostics, Milan, Italy), and absorbance was read at 490 nm using a multiplate reader. In this assay, *Lacticaseibacillus rhamnosus* KCTC5033 (purchased from the Korean Collection for Type Cultures, KCTC, Jeongeup, Republic of Korea) and *Escherichia coli* NCCP 14538 (purchased from the National Culture Collection for Pathogens, NCCP, Cheongju, Republic of Korea) were used as reference strains, with lysis buffer serving as the positive control.

### 2.19. D-Lactate Production Test

The production of D-lactate by LP51 was assessed using a D-lactate colorimetric assay kit (Abcam, Waltham, MA, USA) [29]. The probiotic strain was cultured in an anaerobic incubator for 24 h, after which a reaction sample was prepared according to the manufacturer’s instructions, including the standard curve. To be specific, LP51 culture filtrates corresponding to those containing 1 × 10^7^, 1 × 10^8^, and 1 × 10^9^ CFU were processed, respectively. The reaction mix was then added to the test samples and incubated for 30 min. D-lactate production was measured at 450 nm using a multiplate reader. In this assay, *L. rhamnosus* KCTC5033 (purchased from the Korean Collection for Type Cultures, KCTC, Jeongeup, Republic of Korea) was used as a negative control.

### 2.20. Hemolytic Activity and Bile Salt Deconjugation Test

The hemolytic activity of LP51 was assessed following the protocol of the American Society for Microbiology (ASM) [30]. The strain was streaked onto blood agar (MB Cells, Seoul, Republic of Korea) and incubated for 48 h at 37 °C under anaerobic conditions. Hemolysis was evaluated by observing the zones around the bacterial cultures: complete hemolysis (β), partial hemolysis (α), or no hemolysis (γ). In this assay, *L. rhamnosus* KCTC5033 and *S. aureus* NCCP 14780 were used as reference strains.

The ability of LP51 to deconjugate bile salts was tested following a previously established protocol [31]. The probiotic strain was streaked onto agar plates containing taurodeoxycholic acid (TDCA) sodium salt (Sigma-Aldrich, St. Louis, MO, USA) and incubated at 37 °C under anaerobic conditions for 5 days. Plates were then examined to determine the strain’s bile salt deconjugation ability, with *L. rhamnosus* KCTC5033 serving as the control strain.

### 2.21. Antibiotic Susceptibility Test (E-Test)

An E-test was conducted to assess the antibiotic susceptibility of the candidate probiotic strain, as recommended by the European Food Safety Authority (EFSA) for safety evaluation [32]. A bacterial suspension was inoculated onto Mueller–Hinton agar plates. E-test strips (Liofilchem, Roseto degli Abruzzi, Teramo, Italy), impregnated with a gradient of various antibiotics (ampicillin, vancomycin, gentamicin, kanamycin, streptomycin, erythromycin, clindamycin, tetracycline, and chloramphenicol), were placed on the agar and incubated for 48 h at 37 °C under anaerobic conditions. The minimum inhibitory concentration (MIC) that completely inhibited bacterial growth was determined and compared to the EFSA’s MIC cut-off criteria.

### 2.22. Analysis of SCFAs Using GC-MS: Sourcing and Sample Treatment

Acetic acid (CAS no. 64-19-7, 99.9%), propionic acid (CAS no. 1979-09-4, 99.9%), and butyric acid (CAS no. 107-92-6, 99.7%) were sourced from TCI, while valeric acid (CAS no. 109-52-4, 99.4%) was obtained from Sigma-Aldrich. Sodium chloride (CAS no. 7647-14-5) and sulfuric acid (CAS no. 7664-93-9) were supplied by Daejeong (Seoul, Republic of Korea). In a vial, 2.5 g of NaCl, 5 mL of sample, and 1 mL of 2% sulfuric acid were prepared for analysis using a gas chromatography–mass spectrometry system (Perkin Elmer Clarus 690 GC, Clarus SQ8-GC-MS, TurboMatrix Headspace). Standard solutions were similarly pretreated, with the concentrations for acetic acid diluted to 1, 2, 5, 10, 20, 50, 100, 200, 500, and 1000 mg/L, while propionic acid, butyric acid, and valeric acid were diluted to 1, 2, 5, 10, 20, 50, and 100 mg/L and analyzed under the same conditions.

### 2.23. Ethical Approvals

The tests involving human subjects (patch tests and clinical evaluations) were approved by the Institutional Review Board of Soonchunhyang University (IRB 202303-BR-023-02). The studies were conducted by the Skin Clinical Evaluation Research Institute of the University (study no. S23004). Written consent was obtained from all participants prior to the start of the study.

## 3. Results

### 3.1. Culturomics Results for Isolating Strains from Healthy Female Vaginal Fluid

In this study, we used a culturomics-based technique with various agar media to isolate *Lactobacillus* strains from healthy vaginal fluid. As a result, bacterial growth was observed in 9 out of 30 vaginal samples, from which we isolated 72 bacterial colonies displaying lactic-acid-bacteria-like characteristics (Table S1). The isolated strains were identified using 16S rRNA gene sequencing. Among the selected colonies, 34 isolates were identified as belonging to the *Lactobacillus* genus (Table S2), while the remaining strains were classified as non-*Lactobacillus* (Table S3). The candidate strain LP51 in this study was found to have a 99% similarity to the 16S rRNA sequence of *L. rhamnosus* strain JCM 1136 based on 16S rRNA gene-sequencing analysis (Table 1). Additionally, the sequence showed 97–99% similarity to various species within the *Lacticaseibacillus* genus. Based on these results, it was concluded that the candidate strain belongs to the *Lactobacillus* genus, with further details confirmed through whole-genome sequencing analysis.

### 3.2. Analysis of Whole-Genome Sequencing Results of the Strain

**Figure 1:**
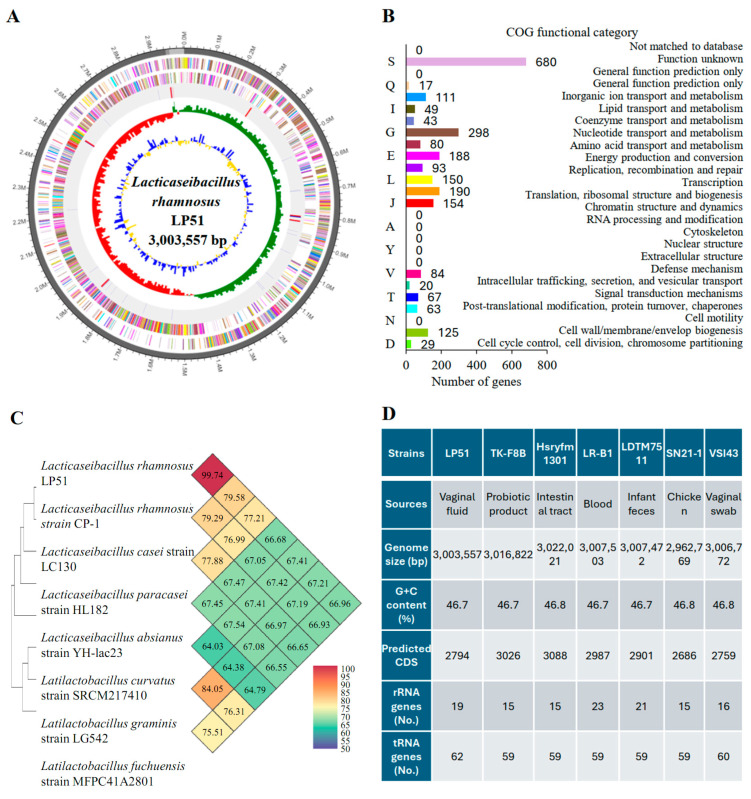
Analysis of whole-genome sequencing results of candidate strain LP51. (**A**) The genome of the strain revealed a single circular chromosome with a length of 3,003,557 bp. The antisense and sense strands, color-coded according to COG categories, are displayed from the outer edge to the center. The circular map also shows tRNA and rRNA, indicated by red and blue, respectively. The inner circles of the map represent the GC skew, with yellow and blue indicating positive and negative values, and the GC content is highlighted in red and green. (**B**) Functional classification of the genes revealed the relative abundance of 2441 proteins assigned to COG (Clusters of Orthologous Groups) families. Biological functions were identified for 1761 proteins, while the roles of 680 CDSs remain unknown. (**C**) The genome similarity of LP51 was analyzed using the OrthoANI (Orthologous Average Nucleotide Identity) method and compared to other *Lacticaseibacillus* and non-*Lacticaseibacillus* strains. The data showed that LP51 had a close similarity of 99.74% with *Lacticaseibacillus rhamnosus* CP-1. It exhibited similarities of 79.58%, 77.21%, and 66.68% with other *Lacticaseibacillus* strains, such as LC130, HL182, and YH-lac23, respectively. (**D**) The chromosomal characteristics of different *L. rhamnosus* strains were compared, and the results strongly suggested that the newly discovered strain, LP51, represents a novel strain of *L. rhamnosus*. The NCBI accession number for the candidate strain *L. rhamnosus* LABIO-PMC-51 (LP51) in this study is PRJNA1123863, and the strains used for genome comparison, along with their NCBI RefSeq assemblies, are Hsryfm 1301 (GCF_008727835.1), TK-F8B (GCF_015377485.1), LR-B1 (GCF_004010975.1), LDTM7511 (GCF_017795605.1), SN21-1 (GCF_033802705.1), and VSI43 (GCF_029011275.1).

The primary genomic characteristics of the LP51 strain are illustrated in Figure 1. LP51 contains a single circular chromosome with an average GC content of 46.7% and a genome length of 3,003,557 bp (Figure 1A). We identified 2794 coding sequences (CDSs) in the genome, which were categorized using the Clusters of Orthologous Groups (COG) system (Figure 1B). Of these CDSs, 2441 proteins were assigned to COG families. Biological functions were identified for 1761 proteins, while 680 CDSs corresponded to conserved proteins with unknown functions in other organisms. Additionally, 2855 hypothetical proteins did not match any known proteins in the database. Furthermore, 62 tRNA and 19 rRNA genes were predicted. The accession number of LP51 in the NCBI is PRJNA1123863.

Similarity analysis for strains with similar 16S rRNA sequences was conducted using the OrthoANI method, which leverages LP51’s whole-genome sequencing data (Figure 1C). When compared to all publicly available *L. rhamnosus* genomes, LP51 showed a 99.74% similarity to *L. rhamnosus* CP-1, significantly exceeding the 95% threshold for species delineation [33]. Additionally, LP51 was compared to other strains within the same genus but belonging to different species, including LC130, HL182, YH-lac23, SRCM217410, LG542, and MFPC41A2801. The similarities were 79.5%, 77.2%, 66.6%, 67.4%, 67.2%, and 66.9%, respectively, all below 80%. These results strongly suggest that LP51 is a strain of *L. rhamnosus*.

Moreover, to verify whether our isolated strain is newly discovered, we compared the genomic information of LP51 to other *L. rhamnosus* strains, such as Hsryfm 1301, TK-F8B, LR-B1, LDTM7511, SN21-1, and VSI43 (Figure 1D). Although they belonged to the same species, their source, genome size, G + C content, CDS, rRNA, and tRNA numbers differed. These findings confirm that the LP51 strain is a novel strain of *L. rhamnosus*.

### 3.3. Preparation of MCB and WCB Stocks for LP51 and Optimization in a Laboratory-Scale Fermenter Using FGM

In this study, 20 Master Cell Banks (MCBs) were prepared for each candidate probiotic strain. Subsequently, 50 Working Cell Banks (WCBs) were prepared from each strain’s MCB (Figure S1). The WCBs were then used for the antituberculosis assay. During WCB preparation, viability and integrity were evaluated.

**Figure 2:**
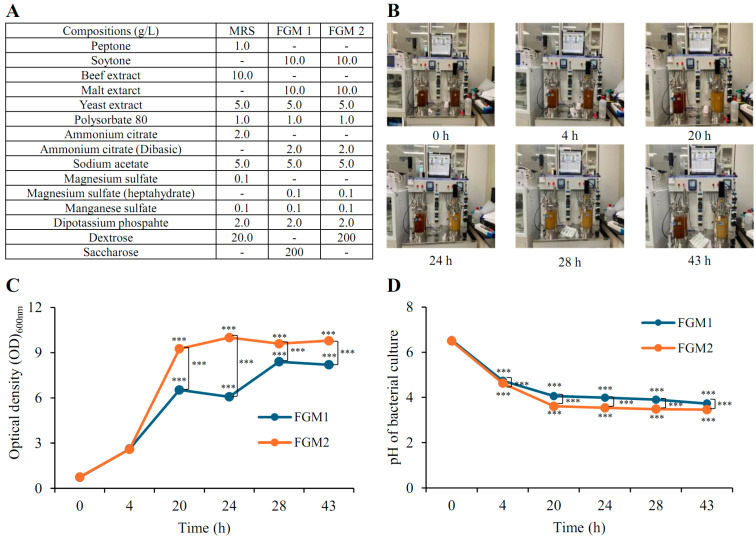
Optimization of LP51 growth in a laboratory-scale fermenter using FGM. (**A**,**B**) The growth ability of the candidate probiotic strain was optimized over 43 h using a laboratory-scale fermenter based on FGM (food-grade medium). (**C**) Bacterial growth was monitored periodically, showing optimal growth in the FGM containing glucose. (**D**) Additionally, pH levels were measured during bacterial growth, revealing lower pH in the glucose medium compared to the sucrose medium. Results are presented as mean values with corresponding standard deviations. Statistical significance between the groups was determined using the Student’s *t*-test (***, *p* < 0.001).

The growth ability of the candidate probiotic strain was optimized using various Food-Grade Medium (FGM), with bacterial growth and pH monitored over time (Figure 2A,B). During the first 4 h of incubation, no significant differences in bacterial growth were observed between the two media (Figure 2C). However, from 20 to 43 h, growth was significantly higher in the FGM2 medium containing glucose compared to that in the FGM1 medium containing sucrose. The pH was consistently and significantly lower in FGM2 compared to that in FGM1 at all measured time points (Figure 2D).

### 3.4. In Vitro Test Results on Skin Hydration Improvement, Skin Barrier Strengthening, and Anti-Atopic, Anti-Inflammatory, Antioxidant, and Cytotoxic Effects

**Figure 3:**
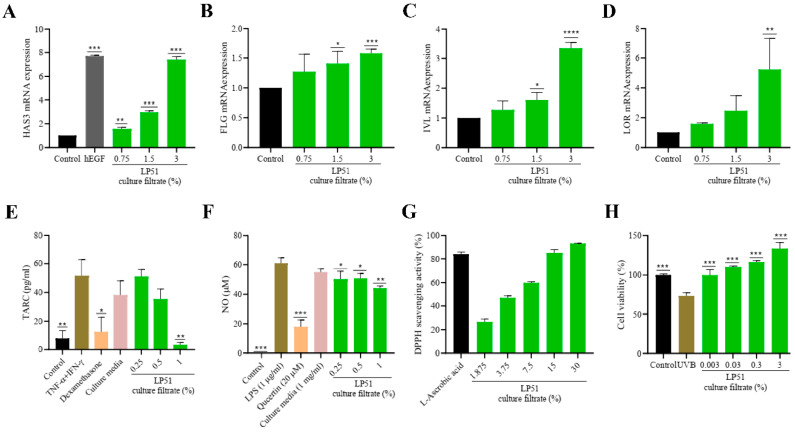
In vitro test results of LP51 culture filtrate on various skin-related parameters. The effects of LP51 on skin hydration, skin barrier strengthening, and anti-atopic, anti-inflammatory, antioxidant, and cytotoxic effects were validated through various in vitro tests. (**A**) The impact of LP51 culture filtrate on skin barrier strength and moisturizing ability was assessed through measurement of *HAS3* (hyaluronan synthase 3) transcription levels in HaCaT (human keratinocyte) cells, showing significant increases at concentrations of 0.75% or higher, with 3% achieving results similar to those of hEGF (human epidermal growth factor, 1 µg/mL). The effect on skin barrier strengthening was evaluated through measurement of (**B**) *FLG* (filaggrin), (**C**) IVL (involucrin), and (**D**) LOR (loricrin) transcription levels in the same cell line, with significant, concentration-dependent increases at 1.5% and 3% LP51. (**E**) The anti-atopic effect was evaluated through an analysis of changes in TARC (thymus and activation-regulated chemokine) production, where 1% LP51 significantly reduced TARC levels in cells treated with TNF-α (10 ng/mL) and IFN-γ (20 ng/mL), demonstrating superior results compared to those of the immunosuppressive corticosteroid dexamethasone (10 µg/mL). (**F**) The anti-inflammatory effect was explored using RAW 264.7 macrophages, where LP51 significantly reduced NO (nitric oxide) levels induced by LPS (lipopolysaccharide, 1 µg/mL) in a concentration-dependent manner, starting at 0.25%. (**G**) LP51 demonstrated strong antioxidant activity, as shown by DPPH (2,2-diphenyl-1-picrylhydrazyl) radical scavenging, with effects similar to those of L-ascorbic acid (50 μg/mL) at concentrations of 15% or higher. (**H**) Additionally, LP51 played a role in restoring cell viability under UVB (ultraviolet B) exposure in HaCaT cells. Results are presented as mean values with corresponding standard deviations. Statistical significance between the groups was determined using the Student’s *t*-test (*, *p* < 0.05; **, *p* < 0.01; ***, *p* < 0.001; ****, *p* < 0.0001).

Various in vitro test results were obtained to assess the effects of the candidate strain LP51 on skin hydration improvement, skin barrier strengthening, and anti-atopic, anti-inflammatory, antioxidant, and cytotoxic effects (Figure 3).

The effect of LP51 culture filtrate on skin barrier strength and moisturizing ability was evaluated by measuring the transcription levels of HAS3 in HaCaT cells (Figure 3A). The results showed a significant increase in HAS3 expression under 0.75% LP51 treatment conditions compared to that in untreated cells. At higher concentrations, the expression levels further increased, reaching the level of expression seen with hEGF (human epidermal growth factor) used as a positive control, especially at the 3% concentration.

Simultaneously, the impact of the candidate strain on skin barrier strengthening was evaluated by measuring the transcription levels of FLG in the same cell line (Figure 3B). The results showed that FLG expression significantly increased in a concentration-dependent manner when treated with 1.5% and 3% PMC-51 culture filtrate. The expression level of IVL was also significantly increased under treatment with the LP51 culture filtrate at a dose level of 1.5% and 3% (Figure 3C). Moreover, the transcription level of LOR was remarkably enhanced under treatment with 3% of LP51 culture filtrate (Figure 3D).

The anti-atopic effect of the candidate strain was also evaluated in the same cell line through an analysis in changes in TARC production (Figure 3E). In cells treated with TNF-α + IFN-γ (positive control), TARC levels significantly decreased in cells treated with 1% LP51 compared to those in untreated cells, showing results superior to those of dexamethasone, an immunosuppressant corticosteroid.

The anti-inflammatory effect of LP51 filtrate was explored using RAW 264.7 macrophages (Figure 3F). Cells treated with LPS showed significant NO production, and when treated with 0.25% or higher LP51 filtrate, NO production significantly decreased in a concentration-dependent manner.

The effect of LP51 on free-radical scavenging was evaluated using DPPH reagent (Figure 3G). The results demonstrated excellent antioxidant effects in a concentration-dependent manner, with the 30% condition showing effects comparable to those of L-ascorbic acid.

The impact of LP51 on UVB protection was assessed using HaCaT cells (Figure 3H). Compared to that in the control group, cell viability significantly decreased when exposed to UVB, but with LP51 treatment at concentrations of 0.003% or higher, cell viability was restored.

### 3.5. Clinical Evaluation of LP51 Formulation in Dry Skin

**Figure 4:**
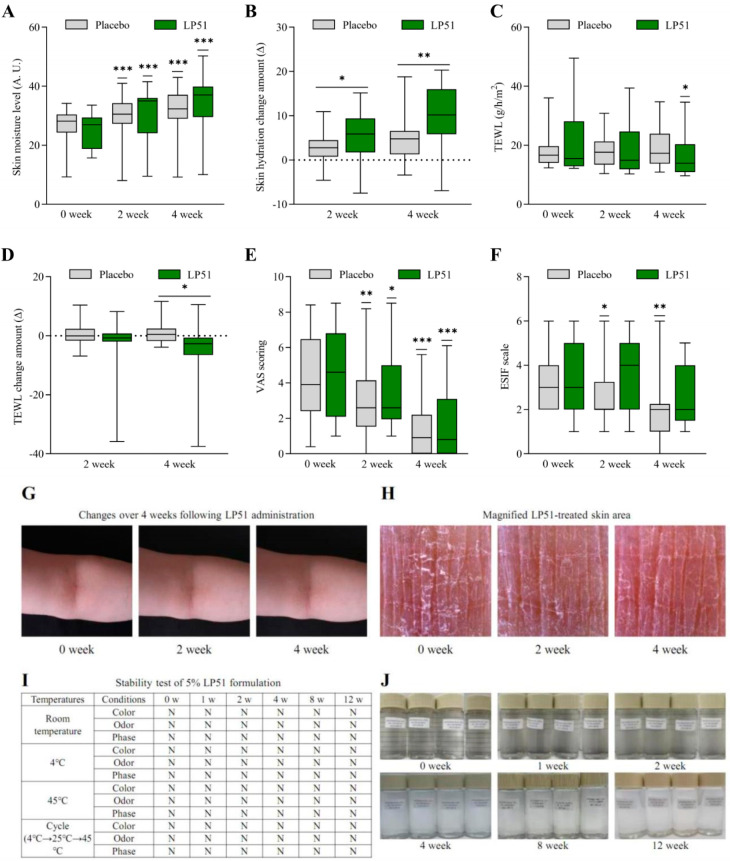
Results of the double-blind clinical trial on the effect of lp51 formulation on dry skin. For efficacy, a 2.9% LP51 formulation was applied to the flexor area of the arms twice daily (morning and evening) for 4 weeks. Evaluations were performed at baseline (week 0), week 2, and week 4 and compared with a placebo group that received the formulation without LP51. (**A**,**B**) Skin hydration levels significantly increased at weeks 2 and 4 compared to baseline, with statistically significant increases compared to placebo. (**C**,**D**) Transepidermal water loss (TEWL) significantly decreased at week 4 compared to baseline, with a significant reduction compared to the placebo. (**E**,**F**) The severity of itching, measured by the visual analog scale (VAS), and the visual appearance assessed by the ESIF scale (erythema, scaling, induration, fissures) showed a decreasing trend, although no statistically significant difference was observed compared to the placebo. The results are presented as means with standard deviations, and the statistical significance between groups was determined using repeated measures ANOVA for VAS scores, while the Mann–Whitney U test using delta values was applied for other comparisons (*, *p* < 0.05; **, *p* < 0.01; ***, *p* < 0.001). (**G**,**H**) Photographs of the treatment area were taken at baseline (week 0), week 2, and week 4 using a digital camera and a Folliscope with a 40× magnification lens, and images for all subjects are provided in the Supplementary Materials. (**I**,**J**) The stability of the formulation was tested, and “N” indicates normal, with corresponding images provided to illustrate the results.

The efficacy of the LP51 formulation for dry skin was evaluated through a double-blind clinical trial, and it was compared to a placebo group that received a formulation without LP51 (Figure 4).

For participants treated with the LP51 formulation, skin hydration levels increased from 25.0 before application to 30.6 after 2 weeks and 35.0 after 4 weeks (Figure 4A). This indicates a significant increase of 22.6% (*p* < 0.001) at 2 weeks and 40.1% (*p* < 0.001) at 4 weeks. In the placebo group, skin hydration also significantly increased from 26.8 before application to 29.6 at 2 weeks and 31.7 at 4 weeks. However, the increase in the LP51 group was significantly greater than that in the placebo group at both 2 weeks (*p* < 0.05) and 4 weeks (*p* < 0.01) (Figure 4B).

The impact on TEWL was also assessed (Figure 4C). In the LP51 group, TEWL decreased from 20.4 before application to 18.5 at 2 weeks and 16.0 at 4 weeks, with a statistically significant reduction of 21.5% observed at 4 weeks (*p* < 0.05). In contrast, the placebo group showed no significant changes, with TEWL values of 17.7 before application, 18.3 at 2 weeks, and 18.6 at 4 weeks. The reduction in the LP51 group was also statistically significant compared to that in the placebo group (*p* < 0.05) (Figure 4D).

The severity of subjective pruritus was assessed using the visual analog scale (VAS) (Figure 4E). In the LP51-treated group, pruritus scores decreased significantly from 4.3 before treatment to 3.5 at 2 weeks (19.4% reduction, *p* < 0.05) and 1.6 at 4 weeks (62.8% reduction, *p* < 0.001). The placebo group also showed a significant reduction in pruritus scores, from 4.3 before application to 2.9 at 2 weeks and 1.4 at 4 weeks, but there were no significant differences between the groups. Visual appearance, evaluated using the ESIF scale (erythema, scaling, induration, and fissuring), showed a tendency to improve, but there were no statistically significant differences compared to the placebo (Figure 4F).

Photographs were taken with a digital camera at each evaluation point (Figure 4G and Figure S2), and magnified images were captured using a Folliscope (Figure 4H and Figure S3).

To check its stability, the test product was stored for 11 weeks under various conditions: chilled (4 °C), room temperature (23 ± 2 °C), accelerated (45 °C), and a cycle of 4 °C  →  25 °C  →  45 °C (Figure 4I,J). The formulation remained homogeneous without any signs of phase separation. Regarding total color change, no noticeable color change was observed during the storage period. No odor was detected under any conditions throughout the study. These results show that the prepared formulation was physically stable for the 11-week storage period.

### 3.6. Results of LP51-Based Skin Metagenomics Analysis Using the Xerosis-Microbiome Index Compared to Placebo

**Figure 5:**
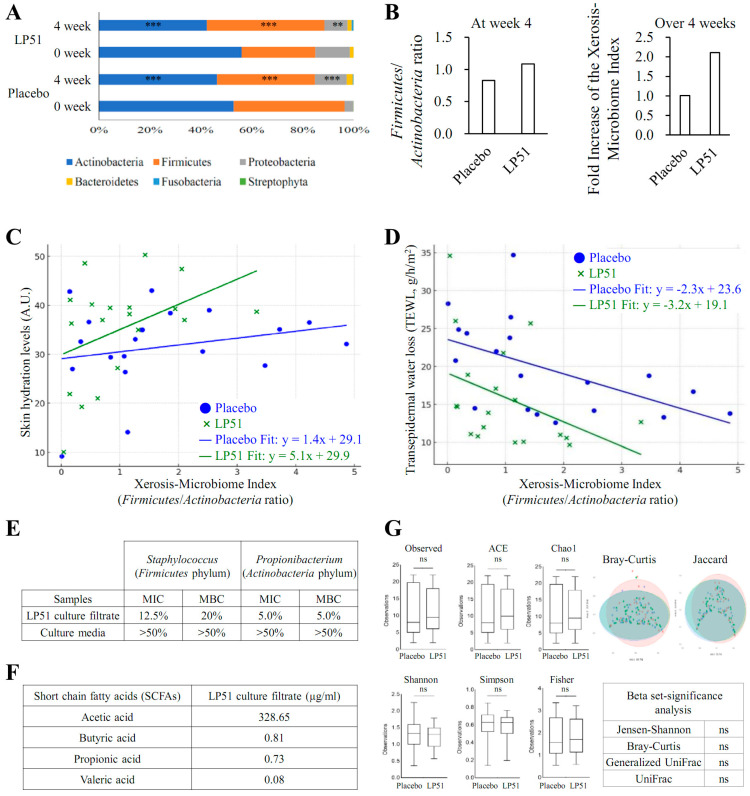
Skin metagenomics analysis based on the xerosis-microbiome index (XMI) for LP51 compared to placebo. (**A**) Over the course of the 4-week clinical trial, skin metagenomic analysis was conducted to examine changes in microbial composition before and after administration. LP51 notably decreased *Actinobacteria* and prominently increased *Firmicutes*. Significant differences in microbial communities were determined using the Wilcoxon signed-rank test (**, *p* < 0.01; ***, *p* < 0.001). (**B**) As a result, LP51 significantly increased the xerosis-microbiome index (XMI), a novel metric proposed in this study based on the *Firmicutes*/*Actinobacteria* ratio, compared to the placebo group. Over the 4-week period, LP51 demonstrated a substantial effect in increasing the fold change of XMI compared to the placebo. (**C**) In the linear regression analysis, XMI showed a positive correlation with skin hydration levels, and LP51 increased the slope of skin hydration relative to XMI. (**D**) Transepidermal water loss (TEWL) exhibited a negative correlation with XMI, and LP51 effectively lowered the y-intercept for TEWL, suggesting a reduction in water loss. (**E**) The in vitro antimicrobial assay results demonstrated that LP51 exhibited superior inhibitory effects on *Propionibacterium acnes* (*Actinobacteria*) compared to *Staphylococcus aureus* (*Firmicutes*), further supporting the observed XMI results. (**F**) The modulation of this XMI is considered to be attributed to the production of short-chain fatty acids (SCFAs) by the LP51 strain. (**G**) Additionally, the alpha and beta diversity analyses indicated that LP51 did not induce dysbiosis in the skin microbiome, showcasing its microbiome-safe properties.

During the 4-week clinical trial, metagenomic analysis was performed on the skin microbiome to observe changes in microbial composition before and after treatment in the target area (Figure 5). LP51 significantly reduced *Actinobacteria* and prominently increased *Firmicutes* (Figure 5A). This shift in microbial balance led to a marked increase in the xerosis-microbiome index (XMI)**,** a ratio of *Firmicutes* to *Actinobacteria*, compared to the placebo group (Figure 5B). Over the 4-week period, LP51 demonstrated a greater fold increase in XMI than placebo, suggesting its effectiveness in improving the microbial balance toward a healthier skin state.

In the linear regression analysis, XMI showed a direct positive correlation with skin hydration levels (Figure 5C). LP51 enhanced the slope of this relationship, meaning that as XMI increased, LP51 amplified the effect on skin hydration more than the placebo did. Conversely, TEWL exhibited an inverse correlation with XMI (Figure 5D). LP51 not only reduced TEWL as XMI increased but also lowered the y-intercept of the regression line, indicating an overall reduction in baseline water loss through the skin.

The results of the in vitro antimicrobial assay revealed that LP51 exhibited a stronger inhibitory effect on *P. acnes* (*Actinobacteria*) compared to *S. aureus* (*Firmicutes*) (Figure 5E). These findings support the above results and further emphasize the role of LP51 in promoting the *Firmicutes*/*Actinobacteria* balance, as reflected in the XMI.

Additionally, the culture filtrate of LP51 was found to contain acetic acid at a concentration of 328.65 µg/mL, butyric acid at 0.81 µg/mL, propionic acid at 0.73 µg/mL, and valeric acid at 0.08 µg/mL (Figure 5F). These SCFAs are considered key regulators in the modulation of XMI.

Lastly, both alpha and beta diversity analyses demonstrated that LP51 did not induce microbial dysbiosis (Figure 5G). Metrics such as observed species, Shannon, Simpson, and Fisher indices for alpha diversity, as well as Bray-Curtis, Jaccard, and UniFrac for beta diversity, showed no significant changes between the LP51 and placebo groups. This indicates that LP51 maintains a stable and healthy skin microbiome, supporting its classification as microbiome safe.

In conclusion, the LP51 treatment significantly modulates the skin microbiome, enhancing beneficial bacteria while maintaining overall microbial diversity. This leads to improved skin hydration and reduced TEWL without causing imbalances in the broader microbiome.

### 3.7. Safety Assessment: Cytotoxicity, D-Lactate Production, Hemolysis, Bile Salt Deconjugation, and Antibiotic Susceptibility

**Figure 6:**
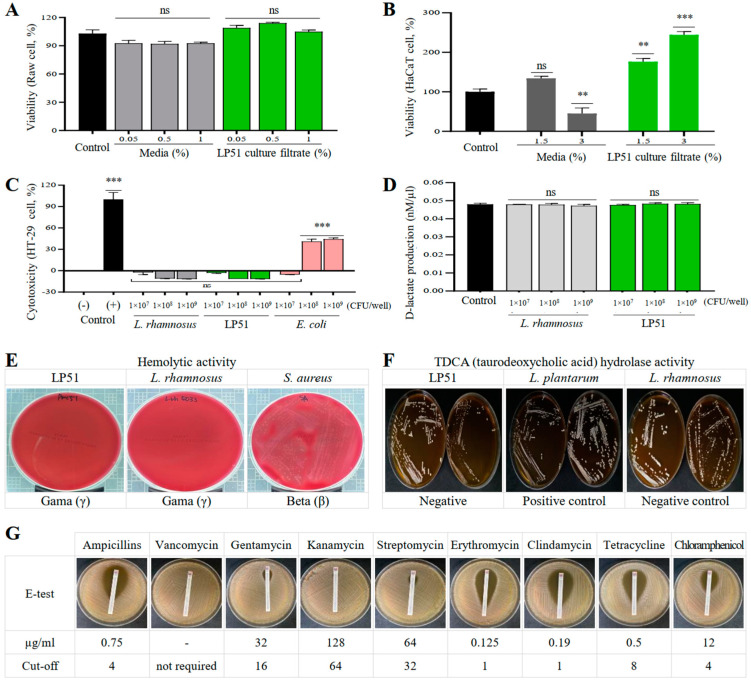
Safety profile of LP51. (**A**–**C**) In the WST-1 assay using mouse macrophagic RAW 264.7 cells and immortalized human keratinocytes HaCaT cells, as well as the LDH assay using human colorectal adenocarcinoma HT-29 cells, LP51 did not exhibit cytotoxicity under any conditions, similarly to *L. rhamnosus* (KCTC 5033). This contrasts with the significant cytotoxicity observed in the positive control (lysis buffer) and pathogenic *E. coli* strain (NCCP 14780). (**D**) Furthermore, D-lactate production did not significantly increase in LP51-treated cells, confirming the safety of LP51, similarly to the aforementioned reference strain *L. rhamnosus*. (**E**) Additionally, LP51 demonstrated γ-hemolysis (no hemolysis) on blood agar, similarly to the *L. rhamnosus* strain mentioned above, in contrast to the β-hemolysis observed with *S. aureus* (ATCC 6538). (**F**) LP51, similarly the reference strain *L. rhamnosus* (KCTC 5033), did not grow on bile salt agar and showed no taurodeoxycholic acid (TDCA) hydrolysis activity unlike the positive control *L. plantarum* (KCTC 3105). (**G**) LP51 was also evaluated for antibiotic susceptibility according to the EFSA (European Food Safety Authority) cut-off criteria. Statistical significance compared to the control group was determined using the Student’s *t*-test (** *p* < 0.01; *** *p* < 0.001).

The safety assessment of the LP51 strain began with the evaluation of cytotoxicity using the water-soluble tetrazolium salt-1 (WST-1) assay and lactate dehydrogenase (LDH) release test. The WST-1 assay using RAW 264.7 cells (Figure 6A) and HaCaT cells (Figure 6B), as well as the LDH test using HaCaT cells (Figure 6C), showed no cytotoxicity at any tested concentration of LP51. In contrast, significant toxicity was observed in the positive controls, which included the lysis buffer and pathogenic *E. coli* strain.

Regarding D-lactate production, LP51 did not show any significant increase at any of the tested concentrations compared to the control and showed similar levels to those observed with the reference *L. rhamnosus* strain (Figure 6D).

Additionally, LP51 exhibited γ-hemolysis (no hemolysis) on blood agar, indicating an absence of β-hemolysis (complete hemolysis) or α-hemolysis (partial hemolysis), similarly to the reference *L. rhamnosus* strain (Figure 6E). In contrast, *S. aureus* displayed hemolytic activity, forming clear zones around its colonies. Moreover, LP51 demonstrated growth in bile-salt-containing agar, indicating bile salt deconjugation activity comparable to that of the reference *L. rhamnosus* strain (Figure 6F).

In terms of antibiotic susceptibility, the LP51 strain was sensitive to antibiotics such as ampicillin (MIC 0.75 µg/mL), erythromycin (MIC 0.12 µg/mL), clindamycin (MIC 0.19 µg/mL), and tetracycline (MIC 0.5 µg/mL) (Figure 6G). However, it showed resistance to gentamicin (MIC 32 µg/mL), kanamycin (MIC 128 µg/mL), streptomycin (MIC 64 µg/mL), and chloramphenicol (MIC 12 µg/mL).

## 4. Discussion

As human skin harbors diverse microbial communities that play a crucial role in both health and disease, there is a growing consensus that dysregulated interactions between the host and these microbial communities can contribute to skin disorders [34]. This has sparked an explosive increase in interest in the skin microbiome, driving ongoing research into various microbiome-based therapeutic approaches for dermatological conditions [34]. Dry skin, often considered a benign but troublesome skin condition, has traditionally been managed using chemical-based treatments, which can have limitations such as skin irritation and limited long-term effectiveness [35]. In this context, this paper proposes a novel approach to addressing dry skin by targeting both the underlying skin barrier dysfunction and the modulation of the skin microbiome through the use of a microbiome therapeutic.

The candidate microbiome therapeutic LP51 in this study is derived from the healthy vaginal microbiome. The establishment of the skin microbiome begins in utero, with colonization occurring through vaginal birth when infants are exposed to typical vaginal bacteria within the first few days of life [36]. Recently, there have been attempts to colonize cesarean-born infants with vaginal bacteria by swabbing them with vaginal secretions [36]. Cesarean section (CS) births are known to affect the development of skin disorders, such as allergic diseases and atopic dermatitis [37]. In this context, we aligned our previous research on the vaginal microbiome of Korean women with the candidate microbiome therapeutic for skin xerosis investigated in this study [13,14]. The strain LP51 was identified as *L. rhamnosus* based on a 98.65% similarity in the 16S rRNA gene sequence and a 95% similarity threshold in whole-genome sequencing [33,38]. Due to its distinct genomic characteristics compared to other *L. rhamnosus* strains, it was confirmed as a novel strain. It should also be noted that *L. rhamnosus* is now classified as *Lacticaseibacillus* [39]. Master and working cell banks (MCB and WCB) were established for the strain [15], and it was optimized using a laboratory-scale fermenter with food-grade medium, suitable for human consumption [40]. A series of detailed analyses were then performed to investigate its effects.

LP51 demonstrated a strong ability to improve skin barrier function by significantly increasing filaggrin protein expression in HaCaT cells. Filaggrins are the key components of the granular cell layer of the epidermis in human skin, and deficiencies in these parts lead to defective skin barrier function [41]. It also showed an enhanced expression of loricrin and involucrin, which facilitate the terminal differentiation of skin cells and thus play a crucial role in maintaining the integrity of skin barrier [42]. In addition, LP51 application enhanced the expression of hyaluronan synthase, an enzyme responsible for synthesizing hyaluronic acid. Hyaluronic acid is highly abundant in the skin and acts as a key molecule involved in skin moisture, with a unique capacity for retaining water [43]. Furthermore, LP51 showed its potential as an anti-atopic agent by reducing TARC levels in HaCaT cells. TARC is a chemokine produced by keratinocytes, endothelial cells, dendritic cells, and other cell types in response to inflammatory signals, and it serves as a marker for skin disorders such as atopic dermatitis [44]. TARC production increases when keratinocytes are treated with pro-inflammatory cytokines such as TNF-α and IFN-γ [45]. In contrast, TARC production decreases when cells are exposed to dexamethasone, a corticosteroid that inhibits TARC production in human keratinocytes [46]. Our results align with the effects of dexamethasone, showing that LP51 can reduce TARC levels, demonstrating its potential as an anti-atopic agent. Additionally, LP51 treatment significantly reduced the generation of nitric oxide (NO) in RAW 264.7 cells, further highlighting the anti-inflammatory effects of our probiotic strain. Studies have shown a close relationship between NO and inflammation [47]. Our findings are consistent with previous research demonstrating the anti-inflammatory effects of probiotics [48]. LP51 also exhibited excellent free-radical-scavenging activity, indicating its antioxidant potential similar to that of L-ascorbic acid, commonly known as vitamin C, which is renowned for its antioxidant properties [49]. This finding is in line with previous studies showing that probiotics can neutralize oxidative stress, protect cells from oxidative damage, and improve skin conditions [50,51,52]. Moreover, the candidate probiotic strain showed a significant ability to protect skin cells from ultraviolet (UV) radiation. Recent studies have highlighted the role of probiotics as natural ingredients that provide photoprotection against UV rays [53,54]. Prolonged exposure to UV rays not only affects the aesthetic appearance of the skin but also damages the normal skin barrier function, increasing the risk of inflammatory skin diseases and even malignancies [55,56].

The clinical efficacy of LP51 as a therapeutic agent for skin disorders was investigated. To achieve this, a skin toner formulation containing the culture filtrate of LP51 was prepared. The formulation demonstrated stability under various temperature conditions and showed no potential for skin irritation in human subjects, aligning with previous findings [54]. Moreover, clinical data revealed a significant improvement in skin moisture content in LP51-treated areas. Skin moisturization plays a crucial role in maintaining normal bodily functions and aesthetic appearance [57]. Conversely, dry skin accelerates aging by reducing elasticity and transforming the skin texture from smooth to rough, making it prone to various skin disorders, thereby diminishing quality of life [58,59]. Studies have shown that probiotics can increase skin moisture content, suggesting their potential as effective moisturizers [59,60]. Additionally, TEWL was significantly reduced after 2 and 4 weeks of treatment. TEWL was used as a key parameter to evaluate the effectiveness of LP51, as it is widely applied to assess epidermal barrier function [61]. Disruptions in skin barrier function are closely related to increased TEWL and have been associated with inflammatory skin conditions such as psoriasis and atopic dermatitis [62]. Furthermore, subjective pruritus levels were measured after treatment with LP51 using the VAS, a commonly used method for assessing pruritus severity [63]. Pruritus, an unpleasant sensation that triggers the desire to scratch, is a major symptom of skin diseases [63,64]. Our findings showed a significant reduction in VAS scores after 2 and 4 weeks of treatment, suggesting the effectiveness of LP51 in alleviating pruritus in dry skin. These results are consistent with previous research showing the beneficial effects of probiotics on pruritus severity [65,66]. A visual assessment was also conducted using the ESIF scale, a widely utilized tool for evaluating skin conditions based on erythema, scaling, induration, and fissuring [67]. No deterioration was observed in any of these four parameters, and the overall ESIF scores decreased after 4 weeks of treatment, indicating both the effectiveness and safety of LP51. Additionally, a post-treatment questionnaire was conducted to assess the product’s safety, after-feel, and overall satisfaction compared to the pre-application period. None of the participants reported issues such as edema, itching, stinging, burning, tightness, prickling, or other adverse symptoms. Overall, our study confirms not only the efficacy of the LP51 formulation but also its safety.

The results presented in this paper provide strong evidence for the therapeutic potential of LP51 in improving skin xerosis by modulating the skin microbiome. During the 4-week clinical trial, metagenomic analysis revealed significant changes in microbial composition, with LP51 notably reducing the abundance of *Actinobacteria* while increasing the proportion of *Firmicutes*. These findings align with previous research examining the relationship between skin moisture levels and microbial communities on the human face [68]. This microbial shift is particularly important as it led to a significant increase in the XMI, a novel metric introduced in this study to quantify the balance between these two major bacterial phyla. The significant rise in XMI in the LP51-treated group compared to the placebo underscores the strain’s efficacy in promoting a healthier skin microbiome, closely associated with improved skin hydration. The linear regression analysis further demonstrated that LP51 not only increased XMI but also enhanced the slope of skin hydration in relation to this index. This indicates that improvements in microbial balance directly contribute to better moisture retention in the skin. Additionally, the negative correlation observed between XMI and TEWL suggests that LP51 effectively reduces water loss, thereby improving skin barrier function in individuals with xerosis. Antimicrobial experiments provided further insights into the mechanisms driving these results. LP51 showed superior inhibitory effects against *S. aureus* (*Firmicutes*) compared to *P. acnes* (*Actinobacteria*), which aligns with the observed shifts in microbial composition and helps explain the improvement in xerosis based on XMI. This modulation of XMI is thought to be associated with the production of SCFAs by LP51. Furthermore, the alpha and beta diversity analyses indicated that LP51 does not induce dysbiosis in the skin microbiome, emphasizing its safety profile. LP51’s ability to regulate key bacterial populations while maintaining microbial diversity demonstrates its microbiome-safe properties, making it a viable candidate for long-term therapeutic use in treating skin xerosis.

The safety profile of LP51 was rigorously evaluated to assess its potential as a therapeutic agent for dermatological disorders. The results demonstrated that LP51 did not exhibit any cytotoxic effects on RAW 264.7 and HaCaT cell lines, aligning with prior studies that underscore its biocompatibility and non-toxic nature [69,70]. Furthermore, the production of D-lactate remained stable even at higher doses, a crucial finding given that elevated D-lactate levels can lead to D-lactic acidosis, which is often associated with conditions such as short bowel syndrome and other gastrointestinal disorders [71]. Maintaining stable D-lactate levels is essential for ensuring the strain’s systemic safety when used as a therapeutic. Additionally, LP51 exhibited susceptibility to antibiotics as per EFSA guidelines, reinforcing its safety in clinical applications, particularly in avoiding the propagation of antibiotic resistance [72]. The strain was also shown to be non-hemolytic, a critical safety marker that mitigates the risk of adverse effects on red blood cells, further validating its safe use in human therapies. Another important aspect of LP51’s safety is its ability to deconjugate bile salts, a property that confirms it does not interfere with normal host physiological processes, thus ensuring that its therapeutic application would not disrupt the host’s gastrointestinal or metabolic functions. Moreover, *L. rhamnosus* has not only been applied in skincare products [6,7] but is also widely recognized for its numerous health benefits, including antimicrobial [27], anticancer [73], antidiabetic [74], anti-obesity [75], and anti-inflammatory properties [76]. These established benefits, alongside its known safety profile, further support the potential of LP51 as a therapeutic agent, emphasizing its strong development potential as a safe and effective treatment.

Recently, with the growing interest in microbiome therapies as next-generation treatments, REBYOTA, a novel microbiome therapeutic for recurrent *Clostridium difficile* infection, became the first of its kind to receive FDA approval in 2022 [77]. Considering the heightened focus on microbiome research for skin disorders, this report on the efficacy of LP51 in alleviating skin xerosis represents a pioneering contribution to the field. In line with FDA guidelines for microbiome therapeutics, LP51 is expanding the foundation of research necessary for its development as a therapeutic agent.

## 5. Conclusions

**Figure 7:**
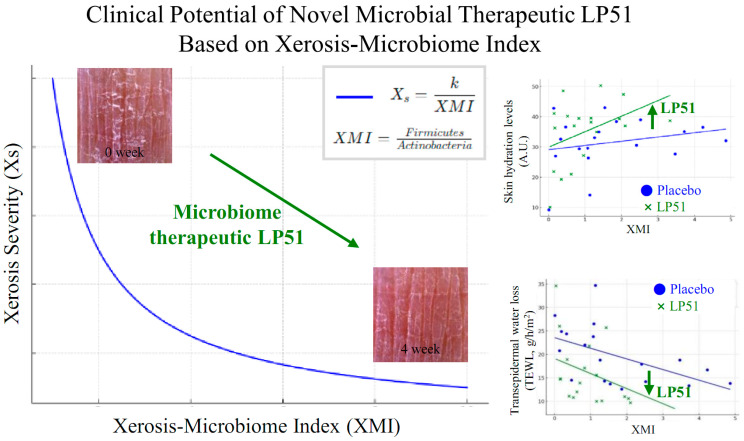
Graphical summary: LP51-mediated modulation of the skin microbiome—enhancing hydration and barrier function according to the xerosis-microbiome index (XMI). This figure presents the key findings of the study, including the effects of LP51 on the skin microbiome, improvements in xerosis, changes in microbial composition, and the correlation between the xerosis-microbiome index (XMI) and skin hydration and barrier function. The summary visually encapsulates how LP51 reduces *Actinobacteria* and increases *Firmicutes*, positively impacting skin hydration and integrity while maintaining microbial diversity and safety. This reinforces the potential of LP51 as a therapeutic agent for treating skin xerosis.

In conclusion, the findings from this study demonstrate the significant potential of microbiome therapeutic LP51 as an innovative treatment for xerosis. The clinical trial results showed that LP51 not only enhanced skin hydration and improved the skin barrier function but also significantly reduced TEWL compared to the placebo group. The additional anti-atopic, anti-inflammatory, and antioxidant properties of LP51 further support its therapeutic benefits, along with its confirmed safety in in vitro cytotoxicity tests. The introduction of the XMI, defined by the *Firmicutes*/*Actinobacteria* ratio, provides a new framework for assessing microbiome-based treatments for dry skin conditions. Collectively, these outcomes highlight LP51 as a promising and safe microbiome therapeutic for effectively addressing xerosis and improving skin health. A graphical summary of this study is presented in Figure 7.

## Figures and Tables

**Table 1:** Identification results based on 16S rRNA gene analysis for candidate strain LP51.

NCBI Ref	Organism	Length	Score	Identity	Gap
NR_043408.1	*Lacticaseibacillus rhamnosus* strain JCM 1136	1521	2641 bits (1430)	1435/1438 (99%)	1/1438 (0%)
NR_037122.1	*Lacticaseibacillus zeae* strain RIA 482	1522	2580 bits (1397)	1424/1438 (99%)	0/1438 (0%)
NR_179712.1	*Lacticaseibacillus chiayiensis* strain BCRC 81062	1536	2575 bits (1394)	1424/1439 (99%)	2/1439 (0%)
NR_113337.1	*Lacticaseibacillus paracasei* strain NBRC 15889	1495	2564 bits (1388)	1421/1438 (99%)	0/1438 (0%)
NR_025880.1	*Lacticaseibacillus paracasei* strain R094	1522	2562 bits (1387)	1421/1438 (99%)	0/1438 (0%)
NR_113333.1	*Lacticaseibacillus casei* strain NBRC 15883	1495	2560 bits (1386)	1421/1439 (99%)	2/1439 (0%)
NR_041893.1	*Lacticaseibacillus casei* DSM 20011	1517	2556 bits (1384)	1422/1440 (99%)	3/1440 (0%)
NR_113823.1	*Lacticaseibacillus paracasei* strain NBRC 15906	1497	2553 bits (1382)	1421/1440 (99%)	2/1440 (0%)
NR_117987.1	*Lacticaseibacillus paracasei* strain ATCC 25302	1441	2551 bits (1381)	1418/1436 (99%)	2/1436 (0%)
NR_115322.1	*Lacticaseibacillus casei* strain BCRC10697	1528	2527 bits (1368)	1419/1443 (98%)	6/1443 (0%)
NR_179363.1	*Lacticaseibacillus mingshuiensis* strain 117-1	1448	2350 bits (1272)	1366/1411 (97%)	7/1411 (0%)
NR_180281.1	*Lacticaseibacillus yichunensis* strain 33-1	1442	2344 bits (1269)	1365/1411 (97%)	7/1411 (0%)

## Data Availability

Data contained in the article and the original data that support the findings of the present study are available from the corresponding author upon reasonable request.

## Supplementary Materials

The following supporting information can be downloaded at https://www.mdpi.com/article/10.3390/cells13232029/s1, Figure S1: Preparation of master cell bank and working cell bank; Figure S2: L Images of the designated arm with test sites after LP51 treatment; Figure S3: Magnified images of test sites following LP51 treatment; Table S1: List of bacterial isolates isolated from vaginal samples of healthy Korean women; Table S2: Identification of *Lactobacillus* spp. based on 16S rRNA gene sequencing; Table S3: List of non-*Lactobacillus* bacterial strains identified in this study.

## References

[1] Walters K.A., Roberts M.S. (2002). The structure and function of skin. Dermatological and Transdermal Formulations.

[2] Archer C.B. (2010). Functions of the skin. Rook’s Textbook of Dermatology.

[3] Harding C.R., Watkinson A., Rawlings A.V., Scott I.R. (2000). Dry skin, moisturization and corneodesmolysis. Int. J. Cosmet. Sci..

[4] Spencer T.S. (1988). Dry skin and skin moisturizers. Clin. Dermatol..

[5] Kamakshi R. (2012). Fairness via formulations: A review of cosmetic skin-lightening ingredients. J. Cosmet. Sci..

[6] Tsai C.C., Chan C.F., Huang W.Y., Lin J.S., Chan P., Liu H.Y., Lin Y.S. (2013). Applications of Spent Culture Supernatant in Cosmetic Antioxidation, Whitening and Moisture Retention Applications. Molecules.

[7] Imko-Walczuk B., Taraszkiewicz A., Mäyrä A. (2019). Soothing Efficacy and Tolerability of a Skin Care Product Containing Live Lactobacillus rhamnosus Bacteria and Berry Seed Oils on Atopic Dermatitis Lesions. J. Cosmet. Dermatol. Sci. Appl..

[8] Grice E.A., Kong H.H., Conlan S., Deming C.B., Davis J., Young A.C., Bouffard G.G., Blakesley R.W., Murray P.R., Green E.D. (2009). Topographical and Temporal Diversity of the Human Skin Microbiome. Science.

[9] Kim K., Jang H., Kim E., Kim H., Sung G.Y. (2023). Recent advances in understanding the role of the skin microbiome in the treatment of atopic dermatitis. Exp. Dermatol..

[10] Gueniche A., Perin O., Bouslimani A., Landemaine L., Misra N., Cupferman S., Aguilar L., Clavaud C., Chopra T., Khodr A. (2022). Advances in Microbiome-Derived Solutions and Methodologies Are Founding a New Era in Skin Health and Care. Pathogens.

[11] Tsai W.H., Chou C.H., Chiang Y.J., Lin C.G., Lee C.H. (2021). Regulatory effects of *Lactobacillus plantarum*-GMNL6 on human skin health by improving skin microbiome. Int. J. Med. Sci..

[12] Catic T., Pehlivanovic B., Pljakic N., Balicevac A. (2022). The Moisturizing Efficacy of a Proprietary Dermo-Cosmetic Product (CLS02021) Versus Placebo in a 4-week Application Period. Med. Arch..

[13] Kim S., Seo H., Rahim M.A., Tajdozian H., Kim Y.S., Song H.Y. (2021). Characteristics of Vaginal Microbiome in Women with Pelvic Inflammatory Disease in Korea. Pol. J. Microbiol..

[14] Kim S., Seo H., Rahim M.A., Lee S., Kim Y.S., Song H.Y. (2021). Changes in the Microbiome of Vaginal Fluid after Menopause in Korean Women. J. Microbiol. Biotechnol..

[15] (2001). ICH Q5D Derivation and Characterisation of Cell Substrates Used for Production of Biotechnological/Biological Products.

[16] Kamioka N., Akahane T., Kohno Y., Kuroki T., Iijima M., Honma I., Ohba M. (2010). Protein kinase C δ and η differently regulate the expression of loricrin and Jun family proteins in human keratinocytes. Biochem. Biophys. Res. Commun..

[17] Blois M.S. (1958). Antioxidant determinations by the use of a stable free radical. Nature.

[18] Budiasih S., Masyitah I., Jiyauddin K., Kaleemullah M., Samer A.D., Fadli A.M., Yusuf E. Formulation and characterization of cosmetic serum containing argan oil as moisturizing agent. Proceedings of the BROMO Conference.

[19] Bolyen E., Rideout J.R., Dillon M.R., Bokulich N.A., Abnet C.C., Al-Ghalith G.A., Alexander H., Alm E.J., Arumugam M., Asnicar F. (2019). Reproducible, interactive, scalable and extensible microbiome data science using QIIME 2. Nat. Biotechnol..

[20] Bacci G., Bani A., Bazzicalupo M., Ceccherini M.T., Galardini M., Nannipieri P., Pietramellara G., Mengoni A. (2015). Evaluation of the Performances of Ribosomal Database Project (RDP) Classifier for Taxonomic Assignment of 16S rRNA Metabarcoding Sequences Generated from Illumina-Solexa NGS. J. Genom..

[21] Chao A., Lee S.M. (1992). Estimating the Number of Classes Via Sample Coverage. J. Am. Stat. Assoc..

[22] Bardenhorst S.K., Vital M., Karch A., Rübsamen N. (2022). Richness estimation in microbiome data obtained from denoising pipelines. Comput. Struct. Biotechnol. J..

[23] Chen Y., Fu X., Ou Z.Y., Li J., Lin S.M., Wu Y.X., Wang X.W., Deng Y.Q., Sun Y. (2023). Environmental determinants and demographic influences on global urban microbiomes, antimicrobial resistance and pathogenicity. NPJ Biofilms Microbiomes.

[24] Lin J.H. (1991). Divergence Measures Based on the Shannon Entropy. IEEE Trans. Inform. Theory.

[25] Beals E.W. (1984). Bray-Curtis Ordination—An Effective Strategy for Analysis of Multivariate Ecological Data. Adv. Ecol. Res..

[26] Kers J.G., Saccenti E. (2022). The Power of Microbiome Studies: Some Considerations on Which Alpha and Beta Metrics to Use and How to Report Results. Front. Microbiol..

[27] Rahim M.A., Seo H., Kim S., Tajdozian H., Barman I., Lee Y., Lee S., Song H.Y. (2022). In vitro anti-tuberculosis effect of probiotic PMC203 isolated from vaginal microbiota. Sci. Rep..

[28] Chen Z.Y., Hsieh Y.M., Huang C.C., Tsai C.C. (2017). Inhibitory Effects of Probiotic on the Growth of Human Colonic Carcinoma Cell Line HT-29. Molecules.

[29] Kang M.S., Yeu J.E., Hong S.P. (2019). Safety Evaluation of Oral Care Probiotics Weissella cibaria CMU and CMS1 by Phenotypic and Genotypic Analysis. Int. J. Mol. Sci..

[30] Buxton R. (2005). Blood agar plates and hemolysis protocols. Am. Soc. Microbiol..

[31] Dashkevicz M.P., Feighner S.D. (1989). Development of a Differential Medium for Bile-Salt Hydrolase-Active *Lactobacillus* spp.. Appl. Environ. Microbiol..

[32] Aquilina G., Bories G., Chesson A., Cocconcelli P.S., de Knecht J., Dierick N.A., Gralak M.A., Gropp J., Halle I., Hogstrand C. (2012). Guidance on the assessment of bacterial susceptibility to antimicrobials of human and veterinary importance. EFSA J..

[33] Aubin G.G., Bemer P., Kambarev S., Patel N.B., Lemenand O., Caillon J., Lawson P.A., Corvec S. (2016). Propionibacterium namnetense sp. nov., isolated from a human bone infection. Int. J. Syst. Evol. Microbiol..

[34] Casterline B.W., Paller A.S. (2021). Early development of the skin microbiome: Therapeutic opportunities. Pediatr. Res..

[35] Simion F.A., Abrutyn E.S., Draelos Z.D. (2005). Ability of moisturizers to reduce dry skin and irritation and to prevent their return. J. Cosmet. Sci..

[36] Schneider A.M., Nelson A.M. (2019). Skin microbiota: Friend or foe in pediatric skin health and skin disease. Pediatr. Dermatol..

[37] Hoel S.T., Wiik J., Carlsen K.C.L., Endre K.M.A., Gudmundsdóttir H.K., Haugen G., Hoyer A., Jonassen C.M., LeBlanc M., Nordlund B. (2023). Birth mode is associated with development of atopic dermatitis in infancy and early childhood. J. Allergy Clin. Immunol. Glob..

[38] Kim M., Chun J. (2014). 16S rRNA gene-based identification of bacteria and archaea using the EzTaxon server. Methods in Microbiology.

[39] Zheng J.S., Wittouck S., Salvetti E., Franz C.M.A.P., Harris H.M.B., Mattarelli P., O’Toole P.W., Pot B., Vandamme P., Walter J. (2020). A taxonomic note on the genus:Description of 23 novel genera, emended description of the genusBeijerinck 1901, and union of and. Int. J. Syst. Evol. Microbiol..

[40] Fenster K., Freeburg B., Hollard C., Wong C., Laursen R.R., Ouwehand A.C. (2019). The Production and Delivery of Probiotics: A Review of a Practical Approach. Microorganisms.

[41] McGrath J.A., Uitto J. (2008). The filaggrin story: Novel insights into skin-barrier function and disease. Trends Mol. Med..

[42] Kim B.E., Leung D.Y.M., Boguniewicz M., Howell M.D. (2008). Loricrin and involucrin expression is down-regulated by Th2 cytokines through STAT-6. Clin. Immunol..

[43] Papakonstantinou E., Roth M., Karakiulakis G. (2012). Hyaluronic acid A key molecule in skin aging. Dermato-Endocrinology.

[44] Kuzumi A., Yoshizaki A., Ebata S., Fukasawa T., Yoshizaki-Ogawa A., Asano Y., Oba K., Sato S. (2021). Serum TARC Levels in Patients with Systemic Sclerosis: Clinical Association with Interstitial Lung Disease. J. Clin. Med..

[45] Sumiyoshi K., Nakao A., Setoguchi Y., Tsuboi R., Okumura K., Ogawa H. (2003). TGF-β/Smad signaling inhibits IFN-γ and TNF-α-induced TARC (CCL17) production in HaCaT cells. J. Dermatol. Sci..

[46] Kurokawa M., Kokubu F., Matsukura S., Kawaguchi M., Ieki K., Suzuki S., Odaka M., Watanabe S., Takeuchi H., Akabane T. (2005). Effects of corticosteroid on the expression of thymus and activation-regulated chemokine in a murine model of allergic asthma. Int. Arch. Allergy Immunol..

[47] Laroux F.S., Pavlick K.P., Hines I.N., Kawachi S., Harada H., Bharwani S., Hoffman J.M., Grisham M.B. (2001). Role of nitric oxide in inflammation. Acta Physiol. Scand..

[48] Rachmilewitz D., Katakura K., Karmeli F., Hayashi T., Reinus C., Rudensky B., Akira S., Takeda K., Lee J., Takabayashi K. (2004). Toll-like receptor 9 signaling mediates the anti-inflammatory effects of probiotics in murine experimental colitis. Gastroenterology.

[49] Gęgotek A., Skrzydlewska E., Litwack G. (2023). Chapter Nine–Ascorbic acid as antioxidant. Vitamins and Hormones.

[50] Briganti S., Picardo M. (2003). Antioxidant activity, lipid peroxidation and skin diseases. What’s new. J. Eur. Acad. Dermatol. Venereol..

[51] Roudsari M.R., Karimi R., Sohrabvandi S., Mortazavian A.M. (2015). Health Effects of Probiotics on the Skin. Crit. Rev. Food Sci..

[52] Kim H., Kim J.S., Kim Y., Jeong Y., Kim J.E., Paek N.S., Kang C.H. (2020). Antioxidant and Probiotic Properties of Lactobacilli and Bifidobacteria of Human Origins. Biotechnol. Bioproc. E.

[53] Bouilly-Gauthier D., Jeannes C., Maubert Y., Duteil L., Queille-Roussel C., Piccardi N., Montastier C., Manissier P., Pierard G., Ortonne J.P. (2010). Clinical evidence of benefits of a dietary supplement containing probiotic and carotenoids on ultraviolet-induced skin damage. Br. J. Dermatol..

[54] Park H.A., Seo H., Kim S., Ul Haq A., Bae S.H., Lee H.J., Ju S.H., Tajdozian H., Rahim M.A., Ghorbanian F. (2024). Clinical effect of PMC48 on hyperpigmented skin. J. Cosmet. Dermatol..

[55] Goukassian D.A., Gilchrest B.A. (2004). The interdependence of skin aging, skin cancer, and DNA repair capacity: A novel perspective with therapeutic implications. Rejuvenation Res..

[56] Orioli D., Dellambra E. (2018). Epigenetic Regulation of Skin Cells in Natural Aging and Premature Aging Diseases. Cells.

[57] Bonté F. (2011). Skin moisturization mechanisms: New data. Ann. Pharm. Françaises.

[58] Proksch E. (2008). The role of emollients in the management of diseases with chronic dry skin. Ski. Pharmacol. Physiol..

[59] Lee D.E., Huh C.S., Ra J., Choi I.D., Jeong J.W., Kim S.H., Ryu J.H., Seo Y.K., Koh J.S., Lee J.H. (2015). Clinical Evidence of Effects of HY7714 on Skin Aging: A Randomized, Double Blind, Placebo-Controlled Study. J. Microbiol. Biotechnol..

[60] Yu J.Y., Ma X.M., Wang X.Y., Cui X.T., Ding K., Wang S.Y., Han C.C. (2022). Application and mechanism of probiotics in skin care: A review. J. Cosmet. Dermatol..

[61] Wikramanayake T.C., Stojadinovic O., Tomic-Canic M. (2014). Epidermal Differentiation in Barrier Maintenance and Wound Healing. Adv. Wound Care.

[62] Green M., Kashetsky N., Feschuk A., Maibach H.I. (2022). Transepidermal water loss (TEWL): Environment and pollution—A systematic review. Ski. Health Dis..

[63] Reich A., Heisig M., Phan N.Q., Taneda K., Takamori K., Takeuchi S., Furue M., Blome C., Augustin M., Stander S. (2012). Visual analogue scale: Evaluation of the instrument for the assessment of pruritus. Acta Derm. Venereol..

[64] Price A., Cohen D.E. (2014). Assessment of pruritus in patients with psoriasis and atopic dermatitis: Subjective and objective tools. Dermatitis.

[65] Boyle R.J., Lahtinen S.J., Tang M.L.K., Pappas A. (2011). Probiotics and Skin. Nutrition and Skin: Lessons for Anti-Aging, Beauty and Healthy Skin.

[66] Matsumoto M., Ebata T., Hirooka J., Hosoya R., Inoue N., Itami S., Tsuji K., Yaginuma T., Muramatsu K., Nakamura A. (2014). Antipruritic effects of the probiotic strain LKM512 in adults with atopic dermatitis. Ann. Allergy Asthma Immunol..

[67] Kumar B., Sandhu K., Kaur I. (2004). Topical 0.25% methotrexate gel in a hydrogel base for palmoplantar psoriasis. J. Dermatol..

[68] Mukherjee S., Mitra R., Maitra A., Gupta S., Kumaran S., Chakrabortty A., Majumder P.P. (2016). Sebum and Hydration Levels in Specific Regions of Human Face Significantly Predict the Nature and Diversity of Facial Skin Microbiome. Sci. Rep..

[69] Du X., Rodriguez J., Wee J. (2022). Dietary Postbiotics Reduce Cytotoxicity and Inflammation Induced by Crystalline Silica in an In Vitro RAW 264.7 Macrophage Model. Foods.

[70] Lee J.Y., Park J.Y., Jeong Y., Kang C.H. (2023). Anti-Inflammatory Response in TNFα/IFNγ-Induced HaCaT Keratinocytes and Probiotic Properties of *Lacticaseibacillus rhamnosus* MG4644, *Lacticaseibacillus paracasei* MG4693, and *Lactococcus lactis* MG5474. J. Microbiol. Biotechnol..

[71] Papagaroufalis K., Fotiou A., Egli D., Tran L.A., Steenhout P. (2014). A Randomized Double Blind Controlled Safety Trial Evaluating d-Lactic Acid Production in Healthy Infants Fed a Formula. Nutr. Metab. Insights.

[72] Yasmin I., Saeed M., Khan W.A., Khaliq A., Chughtai M.F.J., Iqbal R., Tehseen S., Naz S., Liaqat A., Mehmood T. (2020). In vitro Probiotic Potential and Safety Evaluation (Hemolytic, Cytotoxic Activity) of *Bifidobacterium* Strains Isolated from Raw Camel Milk. Microorganisms.

[73] Dehghani N., Tafvizi F., Jafari P. (2021). Cell cycle arrest and anti-cancer potential of probiotic against HT-29 cancer cells. Bioimpacts.

[74] Yadav R., Dey D.K., Vij R., Meena S., Kapila R., Kapila S. (2018). Evaluation of anti-diabetic attributes of MTCC: 5957, MTCC: 5897 and MTCC: 5898 in streptozotocin induced diabetic rats. Microb. Pathog..

[75] Wang T., Yan H., Lu Y.Y., Li X., Wang X., Shan Y.Y., Yi Y.L., Liu B.F., Zhou Y., Lü X. (2020). Anti-obesity effect of LS-8 and MN047 on high-fat and high-fructose diet mice base on inflammatory response alleviation and gut microbiota regulation. Eur. J. Nutr..

[76] Oh N.S., Joung J.Y., Lee J.Y., Kim Y. (2018). Probiotic and anti-inflammatory potential of 4B15 and 4M13 isolated from infant feces. PLoS ONE.

[77] Seo H., Song H.-Y. (2024). Perspectives on Microbiome Therapeutics in Infectious Diseases: A Comprehensive Approach beyond Immunology and Microbiology. Preprints.

