# Supplementary material for: Clinical Potential of Novel Microbial Therapeutic LP51 Based on Xerosis-Microbiome Index
Source: Cells. 2024 Dec 9;13(23):2029. doi: 10.3390/cells13232029 (PMC11639849; doi:10.3390/cells13232029)
Supplement: Supplementary file 1 [file cells-13-02029-s001.zip › cells-3302024-supplementary.pdf]

Supplementary data

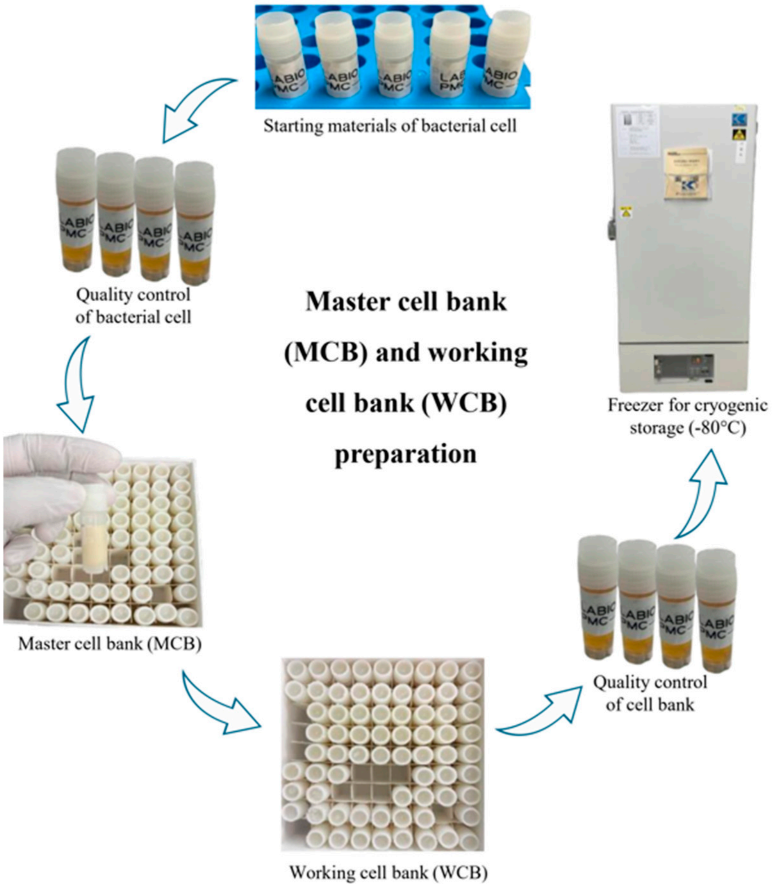

Figure S1. Preparation of master cell bank and working cell bank. After verifying the quality of the isolated strain, a master cell bank was prepared to ensure the integrity of the strain throughout the experimental period. A working cell bank was subsequently prepared from the master cell bank and was frequently used for experiments. All cell stocks were preserved in a deep freezer (-80°C) for long-term storage after quality checks of the prepared cells.

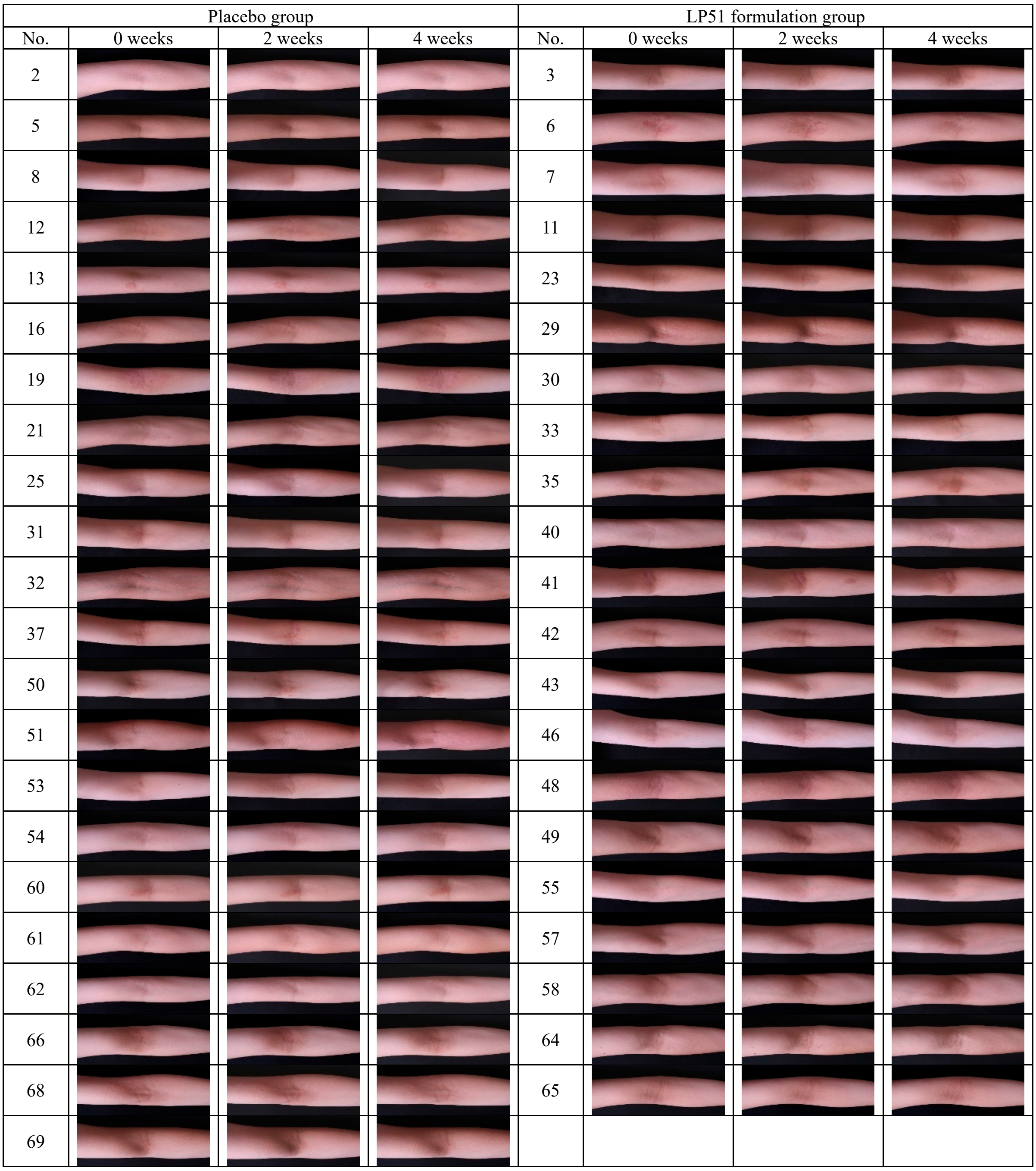

Placebo group				LP51 formulation group			
No.	0 weeks	2 weeks	4 weeks	No.	0 weeks	2 weeks	4 weeks
2				3			
5				6			
8				7			
12				11			
13				23			
16				29			
19				30			
21				33			
25				35			
31				40			
32				41			
37				42			
50				43			
51				46			
53				48			
54				49			
60				55			
61				57			
62				58			
66				64			
68				65			
69							

Figure S2. Images of the designated arm with test sites after LP51 treatment. The formulated product containing LP51 was applied to a designated area of the participants' skin for 4 weeks. Photographs of the treated area were taken using a digital camera at baseline (0 weeks), 2 weeks, and 4 weeks, to demonstrate the effectiveness of LP51.

Placebo group				LP51 formulation group			
No.	0 weeks	2 weeks	4 weeks	No.	0 weeks	2 weeks	4 weeks
2	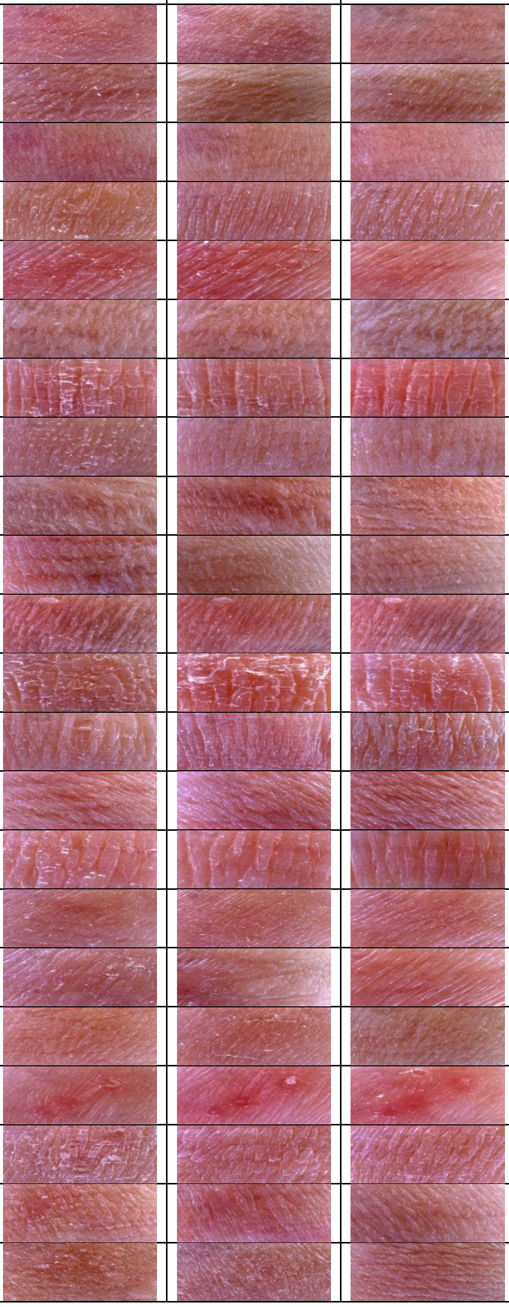			3	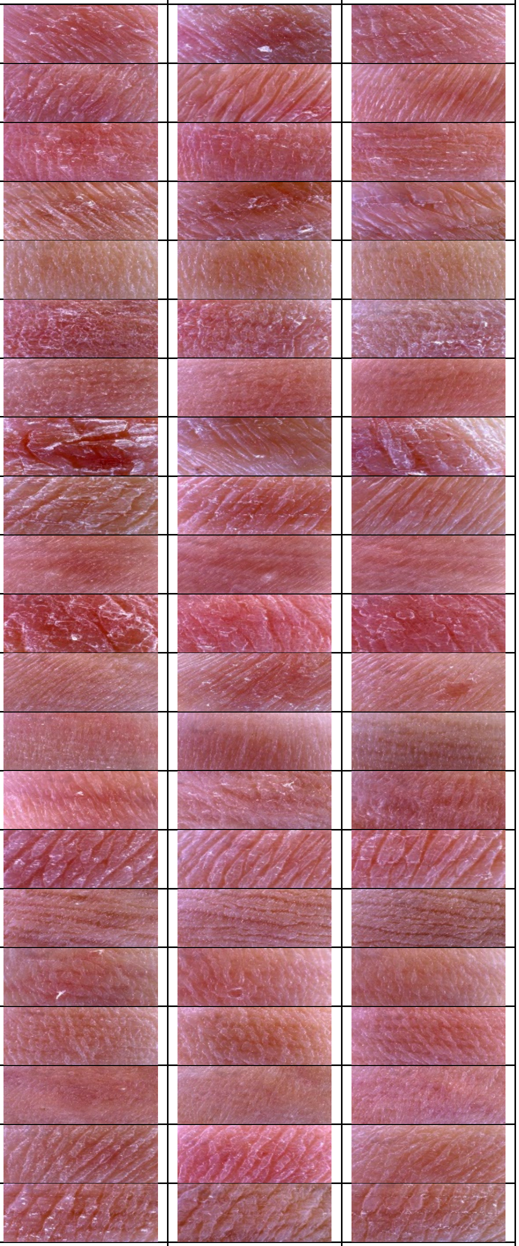		
5				6			
8				7			
12				11			
13				23			
16				29			
19				30			
21				33			
25				35			
31				40			
32				41			
37				42			
50				43			
51				46			
53				48			
54				49			
60				55			
61				57			
62				58			
66				64			
68				65			
69							

Figure S3. Magnified images of test sites following LP51 treatment. The formulated product containing LP51 was applied to a designated area of the participants' skin for 4 weeks. Photographs of the treated area were captured using a folliscope with a 40x magnification lens at baseline (0 weeks), 2 weeks, and 4 weeks to illustrate the effectiveness of LP51.

Table S1. List of bacterial isolates isolated from vaginal samples of healthy Korean women.

Culture condition	Vaginal fluids (VF)								
	VF 05	VF 09	VF 10	VF 13	VF 18	VF 21	VF 26	VF 29	VF 30
MRS agar, Bromocresol purple, and L-cystine, 37°C, Microaerophilic	*05VFBM1, 05VFBM2*	*09VFBM1, 09VFBM2, 09VFBM3*	*10VFBM1, 10VFBM2*	*13VFBM1, 13VFBM2, 13VFBM3*	*18VFBM1, 18VFBM2*	*21VFBM1, 21VFBM2, 21VFBM3, 21VFBM4*	*26VFBM1, 26VFBM2, 26VFBM3*	*29VFBM1, 29VFBM2, 29VFBM3*	*30VFBM1, 30VFBM2, 30VFBM3*
MRS agar, Bromocresol purple, and L-cystine, 37°C, Anaerobic	-	-	-	-	-	-	-	-	
MRS agar, Bromocresol purple, and L-cystine, 37°C, Aerobic	*05VFBA1, 05VFBA2*	*09VFBA1, 09VFBA2*	*10VFBA1, 10VFBA2*	*13VFBA1*	*18VFBA1, 18VFBA2*	*21VFBA1, 21VFBA2, 21VFBA3*	*26VFBA1, 26VFBA2*	*29VFBA1*	*30VFBA1, 30VFBA2*
de Manose Sharpe agar, 37°C, Microaerophilic	*05VFDM1*	*09VFDM1, 09VFDM2*	*10VFDM1*	*13VFDM1, 13VFDM2*	*18VFDM1*	*21VFDM1, 21VFDM2*	*26VFDM1, 26VFDM2*	*29VFDM1, 29VFDM2*	*30VFDM1, 30VFDM2*
de Manose Sharpe agar, 37°C, Anaerobic	-	-	-	-	-	-	-	-	
de Manose Sharpe agar, 37°C, Aerobic	*05VFDA1, 05VFDA2*	*09VFDA1, 09VFDA2*	*10VFDA1*	*13VFDA1, 13VFDA2*	*18VFDA1*	*21VFDA1, 21VFDA2*	*26VFDA1, 26VFDA2*	*29VFDA1, 29VFDA2*	*30VFDA1*

The italics in the table refer to individual isolates.

Table S2. Identification of *Lactobacillus spp*. based on 16S rRNA gene sequencing.

Samples	Isolates	Closest relative of *Lactobacillus spp.*
VF 05	05VFBM1	*Lactobacillus rhamnosus*
	05VFBM2, 05VFDM1	*Lactobacillus gasseri*
VF 09	09VFBM1, 09VFDM1	*Lactobacillus plantarum*
	09VFBM3	*Lactobacillus rhamnosus*
	09VFDM2	*Lactobacillus filtrateum*
VF 10	13VFBM2	*Lactobacillus crispatus*
	13VFBM1	*Lactobacillus acidophilus*
VF 13	13VFBM1	*Lactobacillus rhamnosus*
	13VFDM1, 13VFDM2	*Lactobacillus plantarum*
	13VFBM2, 13VFBM2	*Lactobacillus gasseri*
V 18	18VFBM2, 18VFDM1	*Lactobacillus rhamnosus*
	18VFBM1	*Lactobacillus crispatus*
	18VFBA1	*Lactobacilllus casei*
VF 21	21VFBA2, 21VFDM2	*Lactobacillus plantarum*
	21VFBM1, 21VFBM2	*Lactobacillus rhamnosus*
	21VFBM3	*Lactobacillus paracasei*
VF 26	26VFBM2, 26VFDM2	*Lactobacillus rhamnosus*
	26VFBM1	*Lactobacillus filtrateum*
	26VFBM3	*Lactobacillus sakei*
VF 29	29VFBM1, 29VFDM2	*Lactobacillus casei*
	29VFBM2	*Lactobacillus plantarum*
VF 30	30VFBM2, 30VFBA1	*Lactobacillus rhamnosus*
	30VFBM3	*Pediococcus acidilactici*
	30VFDM1	*Lactobacillus crispatus*

Table S3. List of non-*Lactobacillus* bacterial strains identified in this study.

Samples	Isolates	Closest relatives of non-*Lactobacillus*
VF 05	*05VFBA1, 05VFDA2*	*Enterococcus faecalis*
	05VFBA2, 05VFDA1	*Enterococcus faecium*
VF 09	*09VFBM2, 09VFBA1*	*Enterococcus faecalis*
	09VFBA2, 09VFDA1	*Enterococcus faecium*
	09VFDA2	*Citrobacter baraakii*
VF 10	*10VFBA1, 10VFDM1, 10VFDA1*	*Enterococcus faecalis*
	10VFBA2	*Enterococcus faecium*
VF 13	*13VFBA1*	*Enterococcus faecalis*
	13VFDA1	*Citrobacter baraakii*
	13VFDA2	*Enterococcus faecium*
V 18	*18VFBA2*	*Enterococcus faecalis*
	18VFDA1	*Enterococcus faecium*
VF 21	*21VFBM4, 21VFBA1*	*Enterococcus faecalis*
	21VFDA2	*Citrobacter baraakii*
	21VFBA3, 21VFDM1	*Enterococcus faecium*
	21VFDA1	*Staphylococcus aureus*
VF 26	*26VFBA1, 26VFBA2, 26VFDA1*	*Enterococcus faecalis*
	26VFDM1	*Enterococcus faecium*
	26VFDA2	*Staphylococcus aureus*
VF 29	*29VFBM3, 29VFBA1*	*Enterococcus faecalis*
	29VFDM1, 29VFDA1, 29VFDA2	*Enterococcus faecium*
VF 30	*30VFBM1, 30VFBA2, 30VFDA1*	*Enterococcus faecalis*
	30VFDM2	*Enterococcus faecium*
